# Recurrent evolution of heat-responsiveness in *Brassicaceae COPIA* elements

**DOI:** 10.1186/s13059-016-1072-3

**Published:** 2016-10-11

**Authors:** Björn Pietzenuk, Catarine Markus, Hervé Gaubert, Navratan Bagwan, Aldo Merotto, Etienne Bucher, Ales Pecinka

**Affiliations:** 1 Department of Plant Breeding and Genetics, Max Planck Institute for Plant Breeding Research, Cologne, 50829 Germany; 2 Present address: Department of Plant Physiology, Ruhr-University Bochum, Bochum, Germany; 3 Department of Crop Science, Federal University of Rio Grande do Sul, Porto Alegre, RS 91540000 Brazil; 4 Department of Plant Biology, University of Geneva, Sciences III, 30 Quai Ernest-Ansermet, 1211 Geneva 4, Switzerland; 5 Present address: The Sainsbury Laboratory, University of Cambridge, Cambridge, UK; 6 Present address: Cardiovascular proteomics, Centro Nacional de Investigaciones Cardiovasculares, Madrid, 28029 Spain; 7 UMR1345 IRHS, Université d’Angers, INRA, Université Bretagne Loire, SFR4207 QUASAV, 49045 Angers, France

**Keywords:** *Brassicaceae*, *COPIA*, Evolution, Heat stress, *ONSEN*

## Abstract

**Background:**

The mobilization of transposable elements (TEs) is suppressed by host genome defense mechanisms. Recent studies showed that the cis-regulatory region of Arabidopsis thaliana *COPIA78*/*ONSEN* retrotransposons contains heat-responsive elements (HREs), which cause their activation during heat stress. However, it remains unknown whether this is a common and potentially conserved trait and how it has evolved.

**Results:**

We show that *ONSEN*, *COPIA37*, *TERESTRA*, and *ROMANIAT5* are the major families of heat-responsive TEs in *A. lyrata* and *A. thaliana*. Heat-responsiveness of *COPIA* families is correlated with the presence of putative high affinity heat shock factor binding HREs within their long terminal repeats in seven *Brassicaceae* species. The strong HRE of *ONSEN* is conserved over millions of years and has evolved by duplication of a proto-HRE sequence, which was already present early in the evolution of the *Brassicaceae*. However, HREs of most families are species-specific, and in *Boechera stricta*, the *ONSEN* HRE accumulated mutations and lost heat-responsiveness.

**Conclusions:**

Gain of HREs does not always provide an ultimate selective advantage for TEs, but may increase the probability of their long-term survival during the co-evolution of hosts and genomic parasites.

**Electronic supplementary material:**

The online version of this article (doi:10.1186/s13059-016-1072-3) contains supplementary material, which is available to authorized users.

## Background

Transposable elements (TEs) are ubiquitous components of eukaryotic genomes. Their functions and roles range from DNA parasites, through regulators of gene transcription to facilitators of genome evolution (reviewed in [1, 2]). Together with other types of repeats, TEs comprise 10–80 % of plant genome content and specific families of long terminal repeat (LTR) retrotransposons can reach thousands of copies per genome [1, 3]. Plants evolved several layers of sophisticated epigenetic silencing mechanisms in order to suppress TE activity. Their transcripts are degraded by the post-transcriptional gene silencing (PTGS) pathway, which greatly reduces possible transposition events [4]. In parallel, transcriptional gene silencing (TGS) stably silences TEs by deposition of DNA methylation via RNA directed DNA methylation (RdDM) mechanism (reviewed in [5, 6]). The repressed state is further stabilized by accumulation of specific histone modifications and faithfully transmitted in a DNA replication-dependent manner to the next generations. External or internal factors [7, 8] can lead to transient loss of silencing, but the epigenetic control will be re-established through tissue-specific RdDM activity [9]. In addition to the nimble epigenetic silencing system, entire TEs can be physically removed from the host genome by deletion-biased homologous recombination processes [10].

In spite of the multi-layer amplification barriers, many TE families show signs of recent transpositions [11–13], suggesting that TEs occasionally escape epigenetic surveillance. There is increasing evidence that stress treatments affect chromatin structure and may lead to transposon activation (reviewed in [1, 14]). A possible mechanism was proposed based on the analysis of stress-induced TEs. LTRs of medicago cold-inducible repetitive element (*MCIRE*) retrotransposon contain a putative cold-responsive element (CRE) in alfalfa (*Medicago sativa*) [15]. The CRE is specified by a conserved 5-bp core sequence (CCGAC) typical for C-repeat (CRT)/dehydration-responsive elements (DRE) that are recognized by cold-specific transcription factors (TFs) [16]. LTRs of heat-responsive *COPIA78/ONSEN* (used as synonyms in this study) retrotransposon in *Arabidopsis thaliana* [7, 8, 17], contain a cluster of four nGAAn motifs forming a heat-responsive element (HRE) [18]. During heat stress (HS), the *ONSEN* HRE is bound by heat shock factor A 2 (HSFA2), which triggers its transcriptional activity. This regulation is very specific and greatly independent of TGS control as the loss of decreased DNA methylation 1 (*DDM1*) in mutant plants did not trigger *ONSEN* transcriptional activation [7], in contrast to other typical LTR retrotransposons [19].

Presence of HRE and CRT/DRE motifs in *ONSEN* and *MCIRE*, respectively, suggested that the TEs’ response to stresses may be mediated by specific TF binding motifs. HREs were previously classified into four types based on their structure and, most likely, also activity [20]. The strongest 4P HRE contains at least four adjacent nGAAn motifs and is bound by two HSFA2 trimers. The 3P HRE is bound by a single HSFA2 trimer and represents a moderately responsive HRE. In contrast, gap and step HREs with irregularly and more distantly spaced nGAAn motifs have on average lower HRE activity. Therefore, the HRE composition needs to be considered in order to define the strength of transcriptional response.

Here we identified multiple heat-responsive *COPIA* families in *Arabidopsis lyrata* and *A. thaliana*, two closely related species, using RNA sequencing (RNA-seq). Subsequently, we extended our analysis to five other *Brassicaceae* species and reconstructed putative HREs, their evolutionary history, and validated our predictions by transcriptional analysis after HS treatment.

## Results

### Identification of heat-responsive TE families in *A. thaliana* and *A. lyrata*

**Fig. 1:**
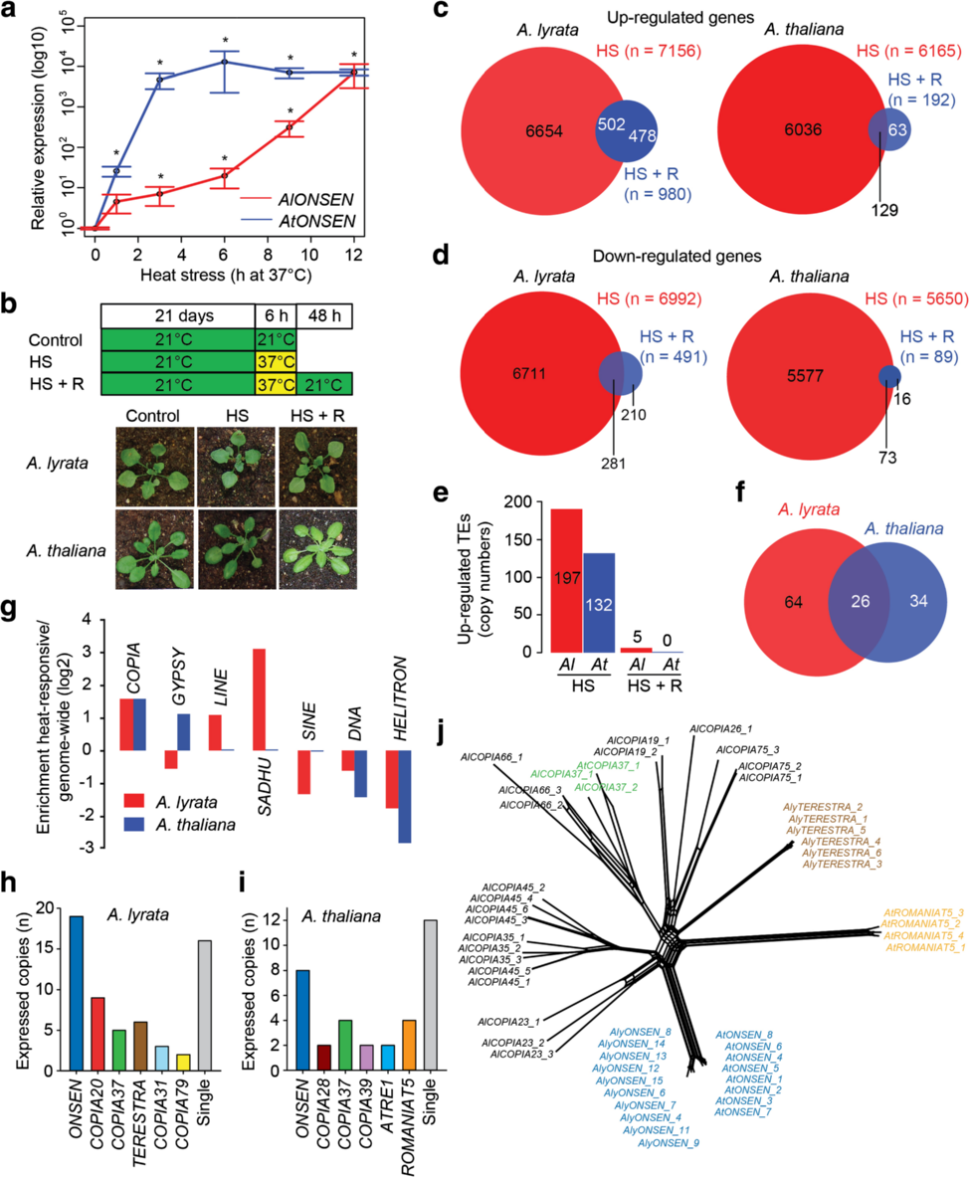
Transcriptome analysis of heat-stressed *A. lyrata* and *A. thaliana* plants. **a** Effects of HS on *ONSEN* heat-responsiveness in *A. thaliana* and *A. lyrata*. Both species were stressed at 37 °C for the indicated number of hours (h) and subsequently analyzed for the amount of *ONSEN* transcript (log10) by RT-qPCR relative to *GAPD-H* transcript amounts. * significant (t-test, *P* <0.05) transcript enrichment relative to 0 h control. *Error bars* indicate standard deviation of three biological replicates. **b** Design of plant HS treatment for RNA-seq and representative phenotypes of control, 6 h heat-stressed at 37 °C and recovered plants. **c**, **d** Number of significantly (**c**) upregulated or (**d**) downregulated protein-coding genes after 6 h at 37 °C and 48 h recovery at non-stress conditions in both species. **e**, **f** Number of significantly upregulated (**e**) TEs and (**f**) TE families after 6 h HS and 48 h recovery. **g** Identification of TE groups enriched for heat-responsive copies. Retrotransposons were divided into *SINE*, *SADHU*, *LINE*, *COPIA*, and *GYPSY* family members. The relative enrichment of heat-activated TEs was calculated as ratio between % of all heat-activated to % of all TEs genome-wide and expressed on a log2 scale. The major heat-responsive *COPIA* families in (**h**) *A. lyrata* and (**i**) *A. thaliana*. The families containing a single HRE are displayed as “single copies.” **j** RT amino acid sequences (Additional file 12)-based phylogenetic network of selected heat-responsive (*colored*) and non-responsive (*black*) *A. lyrata* and *A. thaliana COPIA* families. The data are also provided as un-rooted three in Additional file 5: Figure S2

First, we determined HS conditions that would be effective and comparable for *A. lyrata* MN47 and *A. thaliana* Col-0 plants. As the *A. lyrata* genome contains sequences with high homology to the *A. thaliana ONSEN* retrotransposon, we quantified *ONSEN* transcripts in both species by reverse transcription quantitative polymerase chain reaction (RT-qPCR) during a HS (37 °C) time series using soil-grown plants. Transcripts accumulated faster in *A. thaliana*, but to comparable amounts in both species after 12 h of HS (Fig. 1a). We selected 6 h at 37 °C, leading to a significant and reproducible *ONSEN* transcript accumulation in both species (T-test, *P* <0.05), as the standard HS treatment. Subsequently, samples of control, heat-stressed (6 h HS), and recovered (6 h HS + 48 h 21 °C) plants were RNA-sequenced (Fig. 1b).

To assess the extent of plant responses to HS, we monitored transcript levels from 32,793 *A. lyrata* and 32,678 *A. thaliana* protein-coding genes. This revealed significant upregulation (adjusted *P* <0.05; DESeq) of 21.8 % *A. lyrata* genes (*n* = 7156) and 18.9 % *A. thaliana* genes (*n* = 6165) after 6 h HS (Fig. 1c; Additional files 1 and 2). After recovery, we found only 2.9 % (*n* = 980) of genes still upregulated in *A. lyrata* and 0.6 % (*n* = 192) in *A. thaliana. A. lyrata* showed 21.3 % (*n* = 6992) downregulated genes after HS and 1.5 % (n = 491) after recovery (Fig. 1d). There were 17.3 % (*n* = 5650) significantly downregulated genes after HS and only 0.3 % (*n* = 89) after recovery in *A. thaliana*. Hence, HS treatment induced a similar degree of transient transcriptional changes in both species.

Because there is no publicly available *A. lyrata* TE annotation, we prepared custom-made catalogues of 53,089 *A. lyrata* and 17,009 *A. thaliana* repetitive elements (Additional files 3 and 4, respectively). Although the two species differed threefold in their TEs numbers, their spectra of TE families were similar (Additional file 5: Figure S1). The multi-copy nature of many TEs hinders RNA-seq analysis using standard protocols. Therefore, we developed the *COM*parative *EX*pression of TEs (COMEX) method, which allows quantification of transcripts derived from individual TE copies and effective removal of the RNA-seq reads mapping across TE families (see “Methods;” Additional files 6 and 7). We found 197 and 132 significantly (adjusted *P* <0.05; DESeq) upregulated TEs, representing 90 and 60 families (26 in common), after 6 h HS in *A. lyrata* and *A. thaliana*, respectively (Fig. 1e, f; Additional files 8 and 9). Comparing the major upregulated TE groups versus those in the whole genome revealed general under-representation of *DNA* transposons and *HELITRONs* and *A. lyrata*-specific under-representation of SINEs. In contrast, we found an over-representation of heat-responsive *SADHU* and *LINE* retrotransposons in *A. lyrata*, *GYPSY* elements in *A. thaliana*, and *COPIA* TEs in both species (Fig. 1g). Heat-responsive *AlCOPIAs* (n = 60; 100 %) comprised six families with at least two heat-inducible elements (Fig. 1h): *AlCOPIA31* (*n* = 3; 3 %), *AlCOPIA79* (n = 2; 3 %), *AlCOPIA37* (*n* = 5; 11 %), *AlCOPIA20* (*n* = 9; 14 %), *AlONSEN* (*n* = 19; 37 %), and a so far unknown family which we named **TE**MPERATURE **RES**PONSIVE **TRA**NSPOSON (*TERESTRA*, *n* = 6; 10 %), as well as a bulk of single copies from different families (*n* = 16; 22 %). *A. thaliana* heat-responsive *COPIAs* (*n* = 34) were represented by six families with more than one heat-responsive TE. However, only *AtONSEN* (*n* = 8; 29 %) and *AtCOPIA37* (*n* = 4; 12 %) were common between both species (Fig. 1i). A prominent *A. thaliana*-specific family was *ROMANIAT5*, comprising 12 % (*n* = 4) of all heat-responsive *AtCOPIAs*. After recovery, all TEs were re-silenced in *A. thaliana* and only five (*AlCOPIA37*, *AlRE1*, *SADHU6-1*, *AlATN9_1*, and *AlLINE1_3A*) showed increased transcript amounts in *A. lyrata* (Fig. 1e; Additional file 8). Surprisingly, *ONSEN* was fully silenced after two days of recovery, most likely owing to a shorter HS applied here compared to the previous study [7]. The families representing at least 10 % of heat-responsive *COPIA* elements in each species were considered for further analysis (Fig. 1h).

Next, we tested whether heat-responsive *COPIA* families represent a particular *COPIA* clade. We reconstructed phylogeny of HS-responsive *COPIA37*, *ONSEN*, *TERESTRA*, *ROMANIAT5*, and seven HS-non-responsive *COPIA* families (*19*, *23*, *26*, *35*, *45*, *66*, *75*) based on their RT sequences (Fig. 1j; Additional file 5: Figure S2). The coding sequence was preferred over LTRs for the similarity analysis because this is strongly influenced by length of the input sequences, which may vary drastically in case of LTRs from different families. All heat-responsive families formed distinct and early separated branches, suggesting multiple independent origins of *COPIA* heat-responsiveness.

### The structure and evolution of *ONSEN* heat-responsiveness

**Fig. 2:**
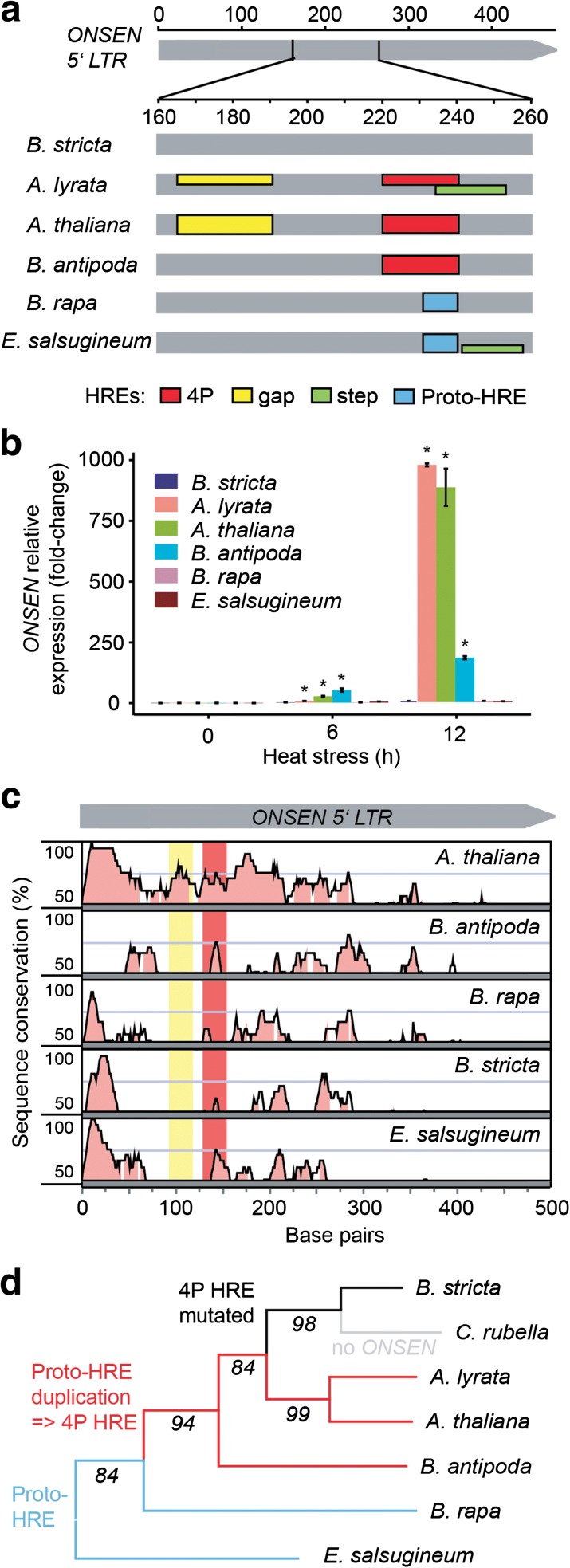
Evolution of *ONSEN* heat-responsiveness. **a** *Schematic representation* of in silico reconstruction of putative HREs in *ONSEN* 5’ LTR in different *Brassicaceae* species. HRE reconstruction follows criteria proposed by [20]. *Colored boxes* spanning the entire height of the *gray field* indicate HREs found in ≥50 % of the heat-responsive copies in *A. thaliana* and *A. lyrata* or all copies in other species. The *lower boxes* represent less frequent (<50 %) variants. Detailed information including sequences underlying individual HREs can be found in Additional file 5: Figure S2. **b** Transcript levels of *ONSEN* elements in *Brassicaceae* after 6 h and 12 h at 37 °C. Quantitative PCR values were obtained using species-specific primer pairs and normalized to *UBC28. Error bars* indicate standard deviation of three biological replicates and * *P* <0.05 in Student’s t-test. **c** Sequence conservation over the *ONSEN* 5’ LTR. Species-specific 5’ LTR consensus sequences were compared to *A. lyrata* query using 20 bp sliding window and 7 bp minimum consensus length. The *y-axis* for each species shows 50–100 % sequence conservation. Regions with ≥70 % similarity (*pink-filled*) were considered as conserved. *Red* and *yellow background colors* indicate the *A. lyrata* 4P and gap HRE regions. **d** Reconstruction of *ONSEN* HRE evolution. The phylogenetic tree was developed using the *CHALCONE SYNTHASE* gene of each individual species. The numbers at branches indicate bootstrap values. *Blue lines* show species with proto-HREs, *red* shows those carrying 4P HREs, *black* shows loss of HREs in *B. stricta*, and *gray* shows the *COPIA78* family in *C. rubella*

There are 24 *COPIA78* elements in *A. thaliana* Col-0 (TAIR10) including eight full-length copies and 16 fragments (Table 1, Additional file 5: Table S1). However, only the eight full-length *ONSEN* copies were found to be heat-responsive (Additional file 9). We performed in silico reconstruction of the putative HREs using a proposed classification [20], which suggested two HREs in all heat-responsive *AtONSENs*: a low efficiency gap HRE and the highest efficiency 4P HRE (Fig. 2a; Additional file 5: Figure S3). While the gap HRE is present in all eight *A. thaliana* full-length *ONSENs*, the 4P was changed into a 3P HRE with moderate efficiency in *AtONSEN4*, due to loss of the fourth motif (Additional file 5: Figure S3). In contrast, none of the 16 fragments or solo LTRs contains functional HREs nor shows heat-responsiveness according to RNA-seq (Additional file 9).

**Table 1:** Copy numbers of elements within analyzed *COPIA* families in *Brassicaceae* species

Species	*ONSEN*		*COPIA37*		*HATE*		*ROMANIAT5*	
	Total	Full	Total	Full	Total	Full	Total	Full^a^
*Arabidopsis lyrata*	55	10	57	5	6	6	131	0
*Arabidopsis thaliana*	24	8	32	1	0	0	49	0
*Ballantinia antipoda*	3	2	0	0	0	0	0	0
*Boechera stricta*	2	2	0	0	14	7	53	0
*Brassica rapa*	6	2	2	0	2	0	7	0
*Capsella rubella*	0	0	2	1	0	0	0^b^	0
*Eutrema salsugineum*	2	1	2	0	6	1	65	0

^a^All *ROMANIAT5* elements lacked integrase domain

^b^Only three solo LTRs were found in *C. rubella*

We found 55 *COPIA78* TEs in *A. lyrata*. Ten are full-length elements and 45 are fragments, either solo LTRs or incomplete according to the gaps in the genome assembly (Table 1; Additional file 5: Figure S3 and Table S2). In total, 15 copies contain at least one putative HRE with three or more adjacent (≤5 bp) nGAAn motifs. Remarkably, a high number of *AlONSENs* carry HREs identical to *A. thaliana* copies (Fig. 2a; Additional file 5: Figure S3). *AlONSEN 2* and *8* have *A. thaliana*-like gap HREs; the 4P type is present in *AlONSEN 10* and both co-occur in *AlONSEN 6*, *7*, *9*, *11*, *14*, *15*, *17*. In addition, we observed putative low efficiency gap/step HREs substituting the 4P HRE in *AlONSEN 1*, *4*, *5*, *12*, and *13*. All *AlONSENs* with predicted 4P HREs were upregulated after 6 h HS (Additional file 8). *AlONSEN 3* and *16* were also found upregulated although they did not contain putative HREs. This was most likely caused by ambiguity in RNA-seq analysis, as 100 % of the reads mapping to these elements were multiply mapping to other *ONSENs*. Hence, there is a high correlation between the predicted HREs and RNA-seq results.

Conservation of the most frequent HRE haplotype between the *Arabidopsis* species raised the question about the evolutionary history of *ONSEN* heat-responsiveness in the *Brassicaceae*. Therefore, we searched for *COPIA78* elements in whole-genome assemblies of *Boechera stricta* v1.2, *Brassica rapa* FPsc v1.3 (both JGI; Phytozome), *Capsella rubella* [21], *Eutrema salsugineum* [22], and low coverage assembly of *Ballantinia antipoda* (Vu, Finke, and Pecinka; unpublished data) using genome-wide BLAST searches. We confirmed the absence of *COPIA78* in *Capsella* [23], but found at least one *ONSEN* copy in all other species (Table 1). RT nucleotide sequence identity was >80 % (Additional file 5: Figure S4), fitting previously proposed criteria for a single TE family [24]. The LTR identity was lower (typically <70 %) due to the presence of insertions and deletions and decreased with phylogenetic distance (Additional file 5: Figure S5). Nevertheless, it allowed us reconstructing putative HREs. None of the other species contained the *A. thaliana*-like gap HRE (Fig. 2a; Additional file 5: Figure S3). However, there was a perfectly conserved 4P HREs in two out of three *ONSENs* in *B. antipoda. ONSENs* of other species either did not contain any HREs (*B. stricta*) or they represented only lower efficiency types and were non-homologous to the *Arabidopsis* HREs (Fig. 2a). To challenge the predicted HREs, we grew all species in vitro and quantified *ONSEN* transcript levels after 6 h and 12 h of HS (Fig. 2b). In agreement with RNA-seq results, we found massive 884–976-fold upregulation in *A. lyrata* and *A. thaliana*. There was also high (185-fold) upregulation in *B. antipoda* containing the putative 4P HRE, but lacking an additional gap HRE (Fig. 2a, b). In contrast, *B. stricta*, *B. rapa*, and *E. salsugineum* predicted to have no or only low efficiency HREs did not show strongly increased *ONSEN* transcript amounts (Fig. 2b).

To test whether HREs represent a major *cis*-regulatory element in *ONSEN* LTRs, we performed phylogenetic shadowing of the LTR consensus sequences (Fig. 2c). Although the 4P HRE region was partially conserved, there are several other similarly conserved regions. The longest stretch of conserved LTR sequence comprises approximately the first 25–30 bp (Fig. 2c), which may be required for TE RT.

By anchoring the structural information on the *Brassicaceae* chalcone synthase-based phylogeny, we reconstructed the evolutionary trajectory of *ONSEN* HREs (Fig. 2d). The nTTCnnGAAn motif, which can be considered as the non-functional sequence preceding the 4P HRE (proto-HRE), is present in *B. rapa* and *E. salsugineum* (Fig. 2a; Additional file 5: Figure S3), suggesting that it existed already at the onset of *Brassicaceae* evolution. Later, proto-HRE became duplicated and instantly created the high affinity 4P HRE. Molecular dating of the split of the *B. antipoda* lineage [25] suggests that this motif was maintained over 6–9 million years of evolution. However, the 4P HRE was occasionally lost due to accumulation of the point mutations (*B. stricta*) or deletion of whole elements (*Capsella*).

### Species-specific gain of HREs in *COPIA37* and the novel family *TERESTRA*

**Fig. 3:**
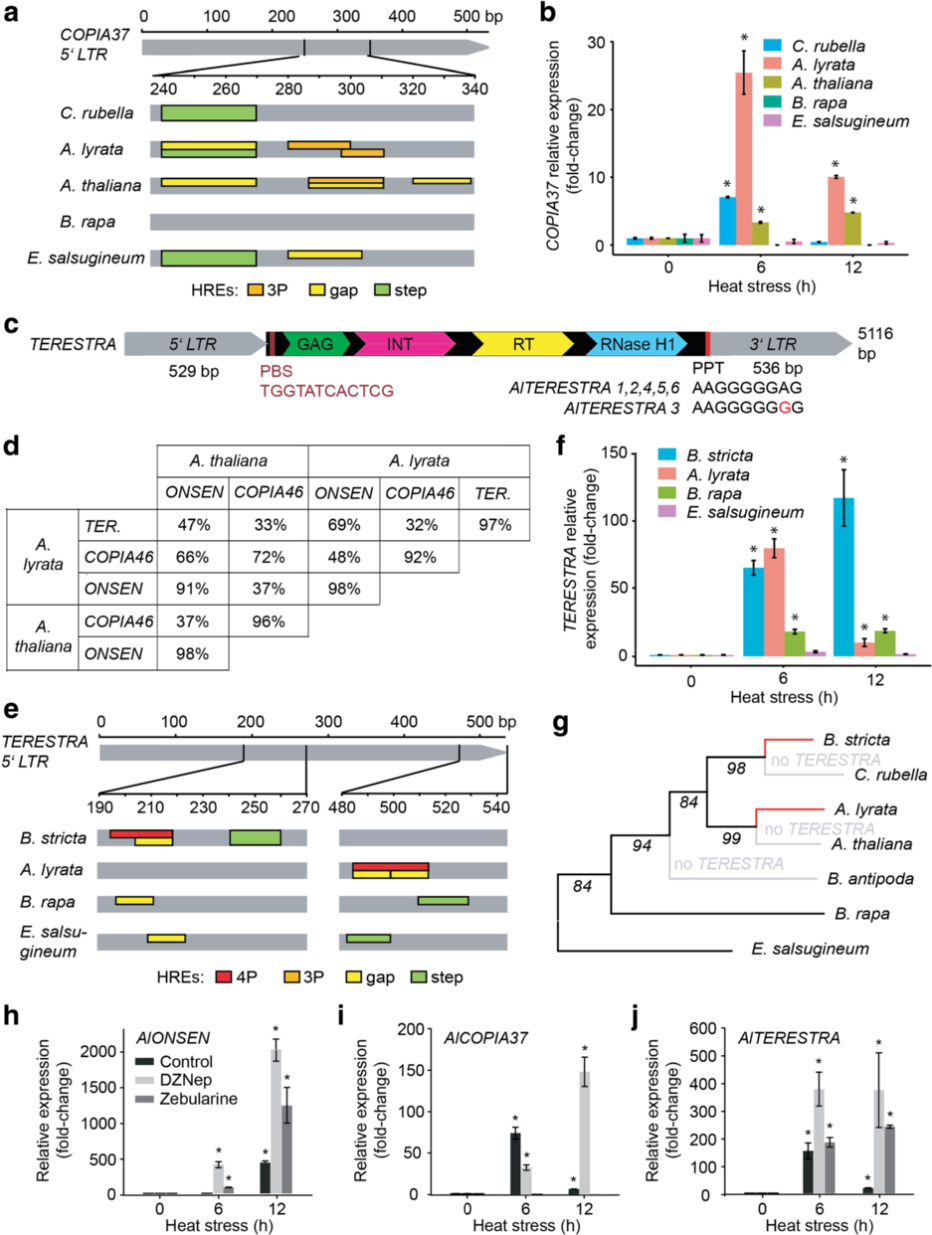
*COPIA37* and *TERESTRA* are novel heat-responsive *COPIA* families. **a** In silico reconstruction of putative HREs in the 5’ LTR of *COPIA37* in different species. HRE classification follows criteria proposed by [20]. *Colored boxes* spanning the entire height of the *gray field* indicate HREs found in ≥50 % of the heat-responsive copies in *A. thaliana* and *A. lyrata* or all copies in other species. The *lower boxes* represent less frequent (<50 %) HREs. Detailed information including sequences underlying individual HREs can be found in Additional file 5: Figure S5. **b** Transcript levels of *COPIA37* in *Brassicaceae* after 6 and 12 h 37 °C HS. The values were normalized to transcript levels of *UBC28. Error bars* indicate standard deviation between three biological replicates and * *P* <0.05 in Student’s t-test. **c** *Schematic representation* of *A. lyrata TERESTRA* (*TERESTRA*). LTRs are indicated in gray. Capsid protein (GAG), integrase (INT), RT, and RNAse H1 domains are shown within the *light-blue-labeled TERESTRA* protein-coding part. Primer binding sequence (PBS) and polypurine tract (PPT) are indicated by *red boxes*. **d** Sequence similarities within pair-wise LTR alignments between *A. lyrata* and *A. thaliana TERESTRA*, *ONSEN*, and *COPIA46* families. More than 70 % similarity was expected for members of the same family. *TERESTRA* is absent in *A. thaliana*. **e** In silico reconstruction of putative HREs in the 5’ LTR of *TERESTRA*. The criteria were as described for Fig. 3a. Detailed information including sequences underlying individual HREs can be found in Additional file 5: Figure S9. **f** Transcript levels of *TERESTRA* in response to 6 and 12 h 37 °C HS in different *Brassicaceae*. The experiment was performed as described in (**b**). **g** Reconstruction of *TERESTRA* HRE evolution. The phylogenetic tree was developed using a chalcone synthase gene of each individual species. The numbers at the base of the branches indicate bootstrap values. *Black lines* show species with low efficiency HREs and *red lines* highlight independently evolved high efficiency HREs in *A. lyrata* and *B. stricta. Gray lines* denote species where *TERESTRA* could not be found. TE transcript accumulation of (**h**) *ONSEN*, (**i**) *COPIA37*, and (**j**) *TERESTRA* after 0, 6, and 12 h 37 °C HS preceded by 48 h control (no inhibitor), 10 μM 3-deazaneplanocin A (DZNep), or 40 μM zebularine treatment. Transcript amounts were normalized to *UBC28* mRNA and signals from drug and heat-treated samples were recalculated as fold-changes relative to 0 h. *Error bars* indicate variation between two biological replicates and * *P* <0.05 in Student’s t-test

The other TE family found to be heat-responsive in both *Arabidopsis* species was *COPIA37* (Fig. 1h, i). However, this phenotype was restricted to fewer copies as only 8.8 % (five out of 57) and 12.5 % (four out of 32) of *A. lyrata* and *A. thaliana COPIA37s*, respectively, showed upregulation upon HS (Additional files 8 and 9). The 5’ LTRs of all heat-responsive copies contained putative low affinity binding gap and step HREs (Fig. 3a; Additional file 5: Figure S6). In addition, we found putative 3P HREs in three *AtCOPIA37s* and two *AlCOPIA37s*. These HREs originated from a common nTTCn rich LTR region, but were not identical. Search in other species revealed the presence of *COPIA37* in *B. rapa* (n = 2), *C. rubella* (*n* = 2), and *E. salsugineum* (*n* = 2; Table 1), but here we found only low affinity binding gap and/or step HREs in the latter two species (Fig. 3a; Additional file 5: Figure S6). To test whether the predicted HREs correlate with heat-responsiveness, we exposed all species to 6 and 12 h HS and quantified the transcript amounts by RT-qPCR (Fig. 3b). We observed up to 25-fold activation for *A. lyrata COPIA37* and a weaker (fivefold) activation for *A. thaliana*, both carrying putative 3P HREs. The amount of *COPIA37* transcript reached its peak at 6 h and decreased in spite of continued HS. Other species, carrying only lower efficiency gap and or step HREs, did either not accumulate the transcript or only at a single experimental point. Hence, the most effective 3P HREs evolved independently in *A. lyrata* and *A. thaliana* and also *COPIA37* elements of other species carry diverse set of HREs.

We also identified *TERESTRA* as a new retrotransposon heat-responsive family. The *A. lyrata* genome contains six *TERESTRA* copies sharing 97 % similarity (Fig. 3d). BLAST searches using *TERESTRA* sequences revealed only local similarities to *ONSEN* LTRs and *COPIA46* GAG and POL domains and no other significant hits. Therefore, we performed de novo *TERESTRA* analysis. Based on the order of GAG and POL, *TERESTRA* was unambiguously identified as *Ty1/COPIA* LTR-retrotransposon (Fig. 3c). Based on only 70 % similarity in an alignment of *TERESTRA* to *COPIA46* and *ONSEN* elements (Fig. 3d), we defined *TERESTRA* as a novel *COPIA* family. The consensus length of the complete *AlTERESTRA* element was 5116 bp and the 5’ and the 3’ LTR were 529 and 536 bp long, respectively (Fig. 3c; Additional file 5: Figure S7). Sequence analyses of *AlTERESTRAs* revealed that all copies are full length, contain a tRNA primer binding site and a polypurine tract, suggesting their autonomy (Fig. 3c). *TERESTRA* LTRs are relatively A-T-rich (69 %) and the consensus sequence contained only a small number of cytosines in symmetrical contexts (CG = 5, CHG = 0; H = A, T or C), which resembles LTR nucleotide composition of *ONSEN* [18]. *TERESTRA* was missing in the *A. thaliana* Col-0 genome. Therefore, we extended our search to 50 *A. thaliana* accessions by genotyping them with *TERESTRA*-specific primers (Additional file 5: Table S3). This screen also gave negative results and suggested the absence of *TERESTRA* in *A. thaliana*. However, we found *TERESTRA* TEs in *Arabidopsis cebennensis* (95 % identity; Additional file 5: Figure S8) and *Arabidopsis halleri* (91 % identity; Additional file 5: Figure S9) using the NCBI sequence database. Furthermore, there were *TERESTRAs* in *B. stricta* (*n* = 14), *B. rapa* (*n* = 2), and *E. salsugineum* (*n* = 6; Table 1), but not outside of the *Brassicaceae*.

All six *AlTERESTRAs* were heat-responsive (Fig. 1h; Additional files 8). Screening of *AlTERESTRA* LTRs for possible HREs revealed a cluster of six nGAAn motifs, which can assemble either two partially overlapping gap HREs or a 4P HRE (Fig. 3e; Additional file 5: Figure S10). Based on the high *AlTERESTRA* transcriptional heat response (Fig. 3f), we favor the latter possibility. Another species with high *TERESTRA* transcriptional activation after 6 h and 12 h HS was *B. stricta* (Fig. 3f). By BLAST we found 14 *TERESTRA* copies in the *B. stricta* genome (Table 1). Eleven copies among them contain a complex cluster of up to five adjacent nGAAn motifs in their 5’ LTRs (Fig. 3d; Additional file 5: Figure S10). According to a conservative approach, three nGAAn motifs within this cluster can putatively form a low affinity gap HRE, but high *TERESTRA* activation in *B. stricta* suggests that all five motifs can establish 4P HREs as compatible with a more relaxed prediction (Fig. 3e, f). Importantly, all predicted *B. stricta* HREs are at positions different from those in *A. lyrata* HREs (Fig. 3e), highlighting their species-specific evolution (Fig. 3g). In *B. rapa*, one *TERESTRA* copy carries a putative gap HRE and another one a step HRE (Fig. 3e). Out of six *TERESTRAs* in *E. salsugineum*, three had putative step and one also an additional gap HRE. However, none of the HREs found in *B. rapa* and *E. salsugineum* was homologous to *Arabidopsis* or *Boechera* HREs and their predicted low HSF binding efficiency was congruent with the absence of heat-responsiveness in the RT-qPCR experiments (Fig. 3f).

Decreased *AlCOPIA37* and *AlTERESTRA* transcript amounts after 12 h versus 6 h HS (Fig. 3b, f) contrasted with continuous transcript accumulation for *AlONSEN* (Fig. 2b). We hypothesized that the failure to maintain high transcript level could be caused by the TGS. Due to a lack of *A. lyrata* TGS mutants, we used a pharmacological approach to interfere with TE silencing [26]. We treated 14-day-old *A. lyrata* plants with 10 μM 3-deazaneplanocin A (DZNep) and 40 μM zebularine, including control plants without treatment. DZNep is an S-adenosylhomocysteine synthesis inhibitor, which blocks the production of SAM, the methyl group donor required for DNA and histone methylation. Zebularine is a cytidine analog leading to DNA de-methylation and loss of silencing from specific transposons [27–29]. After two days of drug treatment, plants were heat-stressed for 0, 6, and 12 h and the amount of transcript analyzed by RT-qPCR (Fig. 3h–j). Control DZNep and zebularine treatment increased *COPIA37* and *ONSEN* transcript tenfold and fivefold, respectively (Additional file 5: Figure S11), suggesting that both TEs can be weakly activated by TGS interference also without HS treatment. *TERESTRA* was not activated by the drug treatment. A combination of HS with drug treatments had strong additive effects in all cases, except for zebularine and HS-treated *COPIA37* (Fig. 3h–j). Both *ONSEN* and *TERESTRA* transcripts accumulated at much higher levels that were not decreasing at 12 h HS (Fig. 3h, i). The effect was generally stronger for DZNep and weaker for zebularine. This suggests that the heat-induced TE transcript accumulation is rapidly suppressed by epigenetic means, in particular for TEs carrying lower affinity binding HREs.

### *AtROMANIAT5* contributes to transcriptional regulation of *APUM9* under HS

**Fig. 4:**
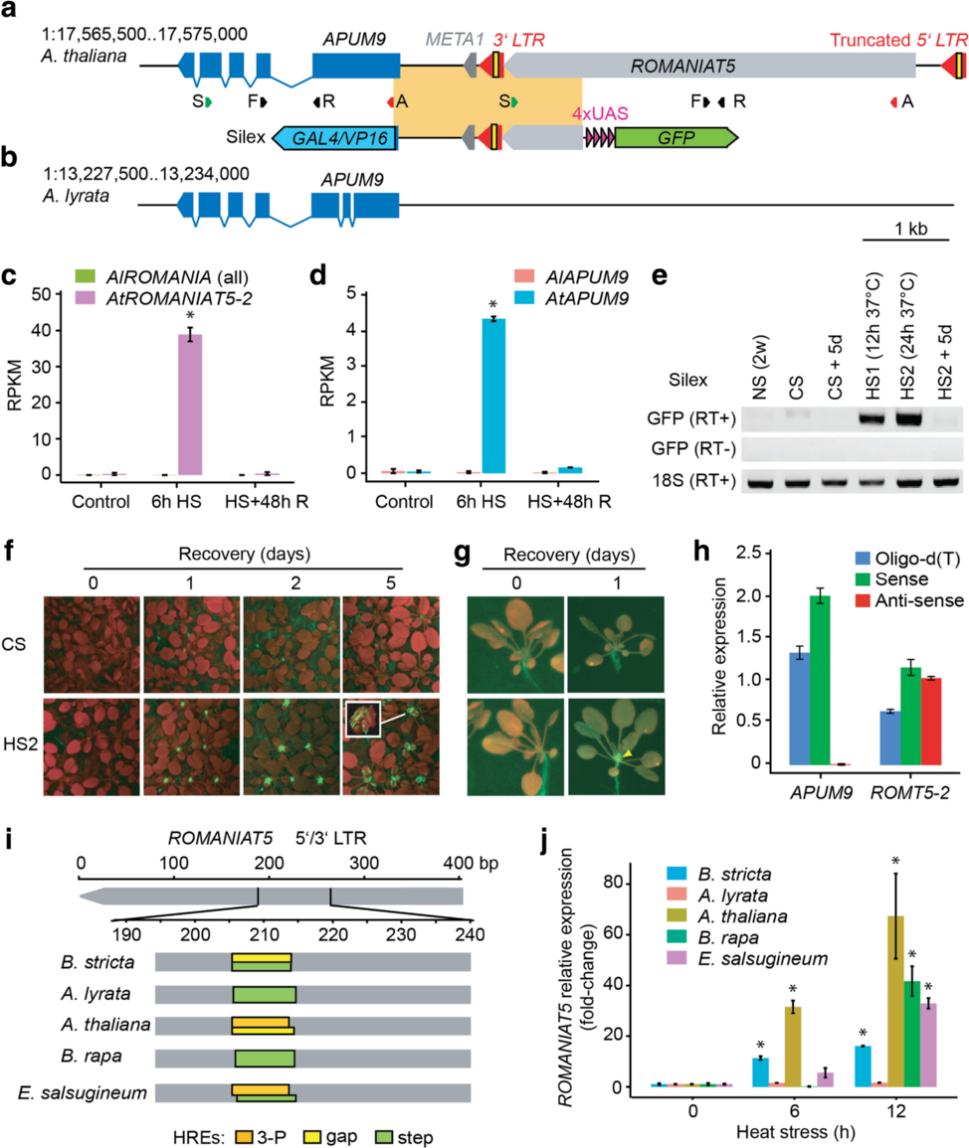
*ROMANIAT5-2* controls heat-responsiveness of *APUM9* in *A. thaliana*. **a** *Schematic representation* of the *ROMANIAT5-2 – APUM9* region in *A. thaliana*. The *yellow block* within the 3’ LTR represents a 3P/Gap heat responsive element (HRE). *S* position of primers for RT of the sense transcripts, *A* position of primers for RT of the anti-sense transcripts, *F* and *R* forward and reverse quantitative PCR primers. *META1* is a transposon fragment flanking *ROMANIAT5-2* 3’ LTR. Silex: the *orange block* corresponds to the genomic fragment cloned upstream of the 4× “upstream activating sequence” (UAS, *violet*) and green fluorescent protein (*GFP*; *green*). **b** *Schematic representation* of the *A. lyrata APUM9* locus. Reads per kilobase per million reads (RPKM) for (**c**) *ROMANIAT5* and (**d**) *APUM9* under control, 6 h at 37 **°**C HS and HS with 48 h recovery at control conditions (HS + R). * *P* <0.05 in t-test. **e** RT-PCR analysis of Silex reporter construct response to HS. *NS* non-stressed control plants, *CS* and *HS* control- or heat-stressed plants, respectively, *+0* and *+5d* days of recovery at non-stress conditions, *RT+* and *RT–* samples with and without RT, respectively. 18S rRNA transcript serves as positive control. **f** GFP signal in control and 24 h heat-stressed (HS2) Silex, detected after 0, 1, 2, or 5 days of recovery. *Red* – chlorophyll, *green* – GFP. **g** *Close-up view* of plants treated as described in (**f**). **h** Strand-specific RT-qPCR of *APUM9* and *ROMANIAT5-2* in *A. thaliana* after 6 h HS. **i** Putative HREs in *ROMANIAT5* LTRs in *Brassicaceae*. **j** RT-qPCR for *ROMANIAT5* in *Brassicaceae* after 6 and 12 h at 37 °C HS. The values were normalized to *UBC28. Error bars* indicate standard deviation between three biological replicates and * *P* <0.05 in Student’s t-test

There are four heat-responsive *ROMANIAT5* TEs in *A. thaliana* but none in *A. lyrata* (Fig. 1h, i; Additional files 8 and 9). All *AtROMANIAT5* elements lack an integrase domain, suggesting that these elements are incomplete and non-autonomous (Table 1). A previous study revealed that one of the heat-responsive copies *AtROMANIAT5-2* (At1g35735) is under complex epigenetic control by Morpheus molecule 1 (*MOM1*) and RdDM pathways, and loss of this control causes upregulation of the *Arabidopsis PUMILIO9* (*APUM9*; At1g35730) gene located directly downstream of the TE [30]. To better understand the potential role of *ROMANIAT5* in regulating *APUM9* during HS, we reconstructed their loci in *A. thaliana* and *A. lyrata* (Fig. 4a, b) and also retrieved the number of reads mapping to both loci under different experimental conditions (Fig. 4c, d). Interestingly, we observed significant (t-test, *P* <0.05) upregulation of *APUM9* upon HS in *A. thaliana* but not in *A. lyrata* (Fig. 4c, d), where the nearby *ROMANIAT5* is missing. This suggested that *ROMANIAT5-2* controls *APUM9* transcription under HS. To validate this observation, we used a reporter line (called Silex) which contains the *APUM9* upstream region and the *ROMANIAT5-2* 3’ LTR upstream of a GFP reporter (Fig. 4a) [31]. The Silex reporter construct is silenced during entire *A. thaliana* development, except for developed siliques, but the reporter activity can be restored in the background of *MOM1* RdDM double mutants and histone deacetylase 6 mutants [31]. We grew Silex reporter plants under controlled conditions with and without HS. GFP transcripts were missing in the control plants but present after 12 and 24 h at 37 °C (Fig. 4e). GFP accumulated in the apical meristem after 24 h of HS recovery and remained detectable for at least five days (Fig. 4f, g), although GFP transcript was not present anymore (Fig. 4e). Heat-responsiveness of Silex transgene in the absence of *ROMANIAT5-2* 5’ LTR suggested that the locus may be at least partially controlled by a bi-directional heat-responsive promoter activity of the 3’ LTR. Indeed, we found putative 3P/gap HREs within the 3’ LTRs (and also the 5’ LTRs) of all heat-responsive *AtROMANIAT5* TEs. However, transcription from the 3’ LTR could result in *ROMANIAT5-2* antisense transcript. To test this, we isolated *A. thaliana* RNA after HS and performed complementary DNA (cDNA) synthesis using strand-specific RT primers (Fig. 4a). Control cDNA from RT with oligo-d(T) primers gave signals for both genetic elements (Fig. 4h). Strand-specific RT-qPCR revealed HS-induced sense transcript, but no antisense transcript, for *APUM9*. In contrast, both types of primers resulted in amplification of *ROMANIAT5-2* transcripts, suggesting that it is transcribed in both directions under HS. The distribution of RNA-seq reads did not indicate large amounts of a read through transcription from *ROMANIAT5-2* to *APUM9* (Additional file 5: Figure S12). Altogether, this confirms the 3’ LTR as bi-directional HS-responsive promoter.

We found *ROMANIAT5* elements in genomes of all species except for *B. antipoda* (Table 1). Putative HREs were present in at least some copies of *ROMANIAT5* in all species except for *C. rubella* that contained only solo LTRs. There were step and gap HREs in *A. lyrata*, *B. rapa*, and *B. stricta*, 3P/gap HREs in *E. salsugineum*, and 3P HREs in *A. thaliana* (Fig. 4i; Additional file 5: Figure S13). The predicted HSF binding affinity of individual HREs correlated well with the amount of *ROMANIAT5* transcripts found after 6 and 12 h of HS (Fig. 4j). The only exception was *B. rapa*, which showed 42-fold upregulation after 12 h HS but the analyzed copies carried at most only low affinity step HRE. This could be due to the presence of heat-responsive *ROMANIAT5* copies in the part of the *B. rapa* genome that is not yet assembled.

## Discussion

Transpositions and insertions of TEs may lead to loss of gene functionality [32, 33]. Therefore, TEs activity and mobility are tightly controlled by epigenetic means throughout the entire plant development [5, 6]. On the other hand, new insertions contribute to genome evolution and regulation of gene transcription [2]. Therefore, it was already suggested in the early days of transposon research that, under conditions when diversity of regulatory patterns in a population may provide a better basis for selection, limited transposon activation could be beneficial [34]. However, how occasional TE expression is provoked and how control is regained later is still a matter of debate. There is a rapidly increasing number of reports showing transient TE activation under various stress conditions reviewed in [1]. Therefore, it was hypothesized that stresses may open a window for transpositions. Here, we introduced the *ONSEN* (*COPIA78*) family as a model for understanding TE control and behavior under HS. *ONSEN* shows massive transcriptional upregulation upon HS in *A. thaliana* and new insertions in progenies of heat-stressed Pol IV mutant [7, 8, 17]. The molecular basis of *ONSEN* heat-responsiveness was puzzling until recently, when a typical HSFA2 TF binding HRE was identified in its *cis*-regulatory region [18]. Presence of canonical TF binding motifs in TE promoters was described for *D. melanogaster* and *M. truncatula* [15, 35]. However, the frequency of such activation strategy among TEs was unknown.

We analyzed LTRs of *A. thaliana* and *A. lyrata* heat-responsive *COPIA* TEs *ONSEN*, *COPIA37*, *TERESTRA*, and *ROMANIAT5* for putative HREs. A minimum of three adjacent (<5 bp) nGAAn motifs can form a basal HRE, whose activity will depend on their distance and the total number [20]. Heat-responsive *COPIAs* featured the whole spectrum of HREs ranging from the 4P types in *ONSEN* and *TERESTRA*, through 3P types in *COPIA37* and *ROMANIAT5* to a dozen of variable gap and step HREs in all these families. By comparing predicted HREs with transcriptional data, we conclude that gap and step HREs are mostly not sufficient to trigger HS-induced TE upregulation. This is congruent with their proposed low HSF binding efficiency [36]. Predicted 3P HREs correlated with up to a hundred-fold (*COPIA37*, *ROMANIAT5*) and 4P HREs with up to a thousand-fold (*ONSEN*, *TERESTRA*) transcript accumulation upon HS. This suggests a strong correlation between putative HREs and the transcriptional response of the TEs.

Previously it was shown that the TGS machinery antagonizes the TE activation [7, 17]. We found that the speed of re-silencing during or after HS depends on the HRE type. While *ONSEN*, with the strong 4P HRE, accumulated transcript during entire HS exposure, TEs carrying lower affinity HREs typically showed a maximum transcript amount at 6 h HS and lower levels at 12 h HS. This silencing can be reduced by treatment with DNA methylation inhibitors. Hence, stressed plants take active measures to prevent TE transpositions already during ongoing HS treatment. However, HS-induced TE activation must not always aim at transposition, but can be part of the plant regulome [2]. In *A. thaliana*, we found that heat-responsive *AtROMANIAT5-2* controlled transcription of the *APUM9* gene located downstream of the element. As we did not observe any evidence for high amount of a read-through transcript from *ROMANIAT5-2* towards *APUM9*, we hypothesize that this transcriptional activation may be mediated rather by a specific three-dimensional chromatin organization at this locus. *APUM9* gene was previously shown to be under control by *HDA6* and synergistically by *MOM1* and RdDM pathways, but not *DDM1* and *MET1* [30, 31]. Therefore, *AtROMANIAT5-2* may represent a domesticated transposon with fine-tuned HS-regulated activation, contributing to transcriptional control of *APUM9*.

To challenge the hypothesis that HREs could be beneficial for TE amplification (but not necessarily for the host genome stability), we reconstructed evolutionary trajectories for HREs of *ONSEN*, *COPIA37*, *TERESTRA*, and *ROMANIAT5* in the *Brassicaceae. ONSEN* was not heat-responsive in the early separated lineages represented by *B. rapa* and *E. salsugineum*, because its LTRs contained only one half of the 4P HRE (proto-HRE), which does not constitute a functional HRE. The proto-HRE became duplicated approximately 6–9 millions of years ago [25] and directly formed the present days 4P HRE found in the genus *Arabidopsis* and in the Australian species *B. antipoda*. Hence, *ONSEN* 4P HRE represents an evolutionary conserved *cis*-regulatory element. However, it should be noted that there are several other similarly or even more conserved regions within the *ONSEN* LTR. Whether they represent other TF binding sites and/or enhancers remains currently unknown. Furthermore, the *ONSEN* example shows that even high affinity HREs do not allow a TE to overrule the host genome defense, because their heat-responsiveness was lost in *B. stricta*, and the whole family became vanished from the *C. rubella* genome. In *TERESTRA*, high affinity 4P HREs evolved independently at two different LTR regions in the closely related species *A. lyrata* and *B. stricta*, while 3P HREs of *COPIA37* emerged multiple times from a common nTTCn-rich LTR region. In contrast to *ONSEN*, HREs of these families are evolutionary young and species-specific. Whether they will be evolutionary successful, is an open question, but we speculate this to be the case for *A. lyrata TERESTRA*, where all genomic copies are full length, carry strong HRE, and respond to heat.

At present it is unknown whether higher temperatures in southern latitudes lead to greater amplification of heat-responsive TEs in subtropical relative to temperate zones. Although this is possible, there are also several factors that may act against such correlation. First, southern populations may reduce effects of HS by adaptation and growth at favorable microclimatic and/or temporal conditions [37]. Second, the genomes are subject to purification mechanisms and the higher transposition rate may be opposed by a greater frequency of TE removal [10]. Indeed, HS was shown to increase frequency of DNA sequence removal by a single strand annealing type of homologous recombination in transgenic constructs structurally resembling a LTR retrotransposon [38, 39]. Therefore, the final number of stress responsive TEs per genome may be the result of multiple effects acting in a complex network.

## Conclusions

TEs evolve *cis*-regulatory elements, such as HREs, rapidly and independently in many groups. This may represent a strategy to produce new copies, constantly challenging the host defense system by searching for potential weak points. Successful regulatory elements may become evolutionary conserved and spread by new TE insertions in a self-reinforcing loop. However, these copies will be silenced and frequently removed from the genome. Hence, stress-mediated TE activation is likely not an unequivocal and straightforward winning principle, but rather a necessary strategy to survive under the pressure of the host defense systems. It is also likely that the host genome can benefit to some extent, and in specific cases, from *cis*-regulatory elements spread by TEs.

## Methods

### Plant materials and growth conditions

We used: *Arabidopis thaliana* Col-0 and Silex [31], *Arabidopsis lyrata* subsp. *lyrata* MN47, *Ballantinia antipoda*, *Boechera stricta* ES9, *Brassica rapa* FPSc, *Capsella rubella*, and *Eutrema salsugineum*. Before standard HS experiments, *A. thaliana* and *A. lyrata* seeds were placed on wet soil, stratified for one week at 4 °C, and then grown in a growth chamber (Percival) at 21 °C during the day and 16 °C during the night (16 h light/8 h dark) until plants reached approximately the five-leaves stage. Subsequently, a part of the plants was transferred to 37 °C for 6 h. RNA samples for sequencing were collected from some of the stressed plants and the controls directly after stress. The remaining stressed plants were allowed to recover at control conditions and collected after 48 h. Later, HS and drug-treatment experiments were performed with in vitro grown plants. First, the seeds were surface-sterilized with 8 % sodium hypochlorite for 6 to 12 min, washed with copious amounts of sterile water, dried under sterile conditions, and spread on sterile ½ Murashige-Skoog medium. After one week of stratification at 4 °C, the Petri dishes with seeds were transferred to growth chamber with a long day regime (16 h light/8 h dark) and constant temperature of 21 °C. Plates with rosettes at the pre-bolting stage were then placed in another chamber with 37 °C for 6 h. For combined drug and heat treatments, *A. lyrata* plants were grown as described above, then transferred to plates with no inhibitor, 10 μM DZNep, or 40 μM zebularine (both Sigma-Aldrich) for 48 h and then exposed to 0, 6, or 12 h at 37 °C HS. Aerial plant tissues were harvested immediately after the stress, flash frozen in liquid nitrogen, and stored at –80 °C.

Seeds of the Silex reporter line were sown directly on potting soil and stratified at 4 °C for 48 h. The pots were then placed in a Percival CU-22 L chamber at 21 °C with 12 h light (140 mmol m^−2^s^−1^) and 12 h dark. When the plants turned 14 days old, the pots were placed at 6 °C under the same light conditions for 24 h. At this time, control plants were moved back to the 21 °C chamber while HS plants underwent 24 h HS at 37 °C with light conditions as before. Immediately after the HS treatment, all pots were placed again at 21 °C. Fluorescence pictures of control and HS plants were taken at 0 and after 1, 2, and 5 days of recovery. Fluorescence imaging was performed using an Aequoria dark box with a mounted ORCAII CCD camera (Hamamatsu, Japan).

### Nucleic acids extraction, cDNA synthesis, and RT-qPCR

Genomic DNA was isolated using the Phytopure gDNA Kit (GE Healthcare). Total RNA was isolated with the RNeasy Plant Mini Kit (Qiagen) with an on column DNaseI (Roche) digestion or by the standard Trizol method with additional DNaseI (Thermo Scientific) digestion. cDNA was synthesized from 1 μg total RNA per sample using the Revert Aid H-Minus First Strand cDNA synthesis kit with the oligo-d(T) primer (all Thermo Scientific). For strand-specific RT, total RNA of 6 h HS *A. thaliana* plants was divided into five aliquots which were converted into cDNA using (1) oligo-d(T) primer, *APUM9* (2) sense and (3) antisense transcript primer, and *ROMANIAT5-2* (4) sense and (5) antisense transcript primers. RT-qPCR analysis was performed on three biological replicates with at least two technical replicates in a CFX384 instrument (BIO-RAD) using the SensiMix Plus SyBr Kit (PEQLAB). Expression values were calculated relative to control-treated samples using the standard curve method [40] and normalized using the glyceraldehyde-3-phosphate-dehydrogenase C2 (*GAPC-2*) or the *UBC28* gene with a stable expression under mock, HS, and recovery conditions. Primers used in this study are listed in Additional file 5: Table S4.

### RNA sequencing

One μg total RNA per sample with RIN >8.0 (Agilent Bioanalyzer 2100) was used to construct strand non-specific sequencing libraries with the Illumina TruSeq RNA Library Kit v2 according to the manufacturer’s instructions. Library quality was tested on a Bioanalyzer and high-quality libraries were subsequently sequenced in the 100 bp single-end read mode using a HiSeq 2500 sequencer (Illumina). Adaptor sequences and low quality bases were trimmed and low quality reads were filtered out with the FAST-X toolkit (http://hannonlab.cshl.edu/fastx_toolkit/) using custom-made scripts. Subsequently, reads were mapped to the corresponding reference genome (TAIR 10 genome assembly or *A. lyrata* genome assembly v1.0) using tophat2 [41] with default settings. On average, >15 million sequencing reads per library passed trimming and quality filtering. The numbers of reads mapping to specific genomic positions were retrieved using Qualimap and the latest *A. thaliana* genome annotation TAIR10 and *A. lyrata* genome annotation v2 [42] for genes and custom-made repeat annotations for TEs. The TE data were further processed with COMEX (see below) and data for genes were analyzed directly using the DESeq package in R software [43, 44].

### COMparative EXpression of transposable elements (COMEX)

Accurate quantification of TEs expression using short read sequences is hampered by high similarity of potentially many genomic copies. We developed a simple protocol called COMEX (https://github.com/bpietzenuk/COMEX) that partially overcomes this problem and allows analysis of TE transcription from RNA-seq data. Out of >10 million reads per average sequencing library, 0.12 % and 0.73 % high-quality mappable reads corresponded to TEs within our custom made *A. thaliana* and *A. lyrata*, respectively, TE annotations. This suggests that TE expression analysis using RNA-seq can be made more sensitive by high sequencing depth. The reads were processed via a shell-script that merges the pipeline as follows. First, the binary mapping.bam file is converted into a readable .sam file. Subsequently, ends are printed (ToPrint_end1.py) to the .sam file and mapping errors are removed (Selectnonrepeated1.py). In the following step (Selectmultiplymapped1.py), the output files for the uniquely mapping and the multiply mapping reads are created. The high-quality uniquely mapping (UM) TE reads were directly accepted for expression analysis. High-quality multiply mapping TE reads were analyzed to identify those providing usable information. We classified multi-mapping reads into two categories: (1) informative reads mapping to multiple members of the same TE family (Specifically Multiply Mapped – SMM); and (2) non-informative reads mapping across TE families (Non-specifically Multiply-Mapped – NMM) using the TE annotation gff-file. Reads of the second category were discarded (new_cases1.py). Afterwards, UM and SMM are merged into a single .sam-file and converted into a binary .bam-file. Subsequently, the output file of the COMEX2.0-pipeline (filename.output.final.bam) containing the number of SMM and UM reads from the same TE family was retrieved using a strand non-specific protocol in Qualimap. To avoid a bias by repeated counting of SMM reads, we used the proportional read count method that divides the power of a read by the number of mapped positions. This provided the number of reads per individual TE families and TEs, which were subjected to statistical analysis using the DESeq package in R software [43]. To avoid considering potentially large number TEs with minimal transcriptional changes, which would be later difficult to validate experimentally, we considered only those which had at least 0.55 RPKM in one of the experimental time points.

### In silico sequence analysis

Sequences of interest were extracted from corresponding TE annotation files using bedtools [45]. LTR reconstruction was carried out in LTR-Finder [46] or manually by pairwise and multiple alignments of the 3'end to the 5'ends of TE annotated regions using MUSCLE or multalin with the DNA 5–0 comparison table option. Structural analysis and annotation of *TERESTRA* was performed using LTR Finder and blastx using NCBI non-redundant protein sequences database. LTR_Finder was used in both analyses with the threshold option set to 2.0 using the tRNA database of *A. thaliana* to predict PBS. The LTR length range was set from 100–3500 and the minimum LTR distance was set to 1000. Other parameters were left at default settings. Search for *ONSEN* sequences within genomes of various *Brassicaceae* was done using BLASTN within Phytozome 10 [47, 48]. Hits with a sequence identity of >70 % were extracted and manually investigated. Positive hits with a query coverage <70 % were analyzed manually for sequence similarity with Multialign using the DNA 5–0 comparison table option. The input ONSEN RT and LTR sequences are provided in Additional files 10 and 11, respectively.

### Phylogenetic analysis

To analyze the evolutionary distance of the *Ty1/COPIA* LTR-retroelements, multiple sequence alignments of the RT domains were performed using the genomic nucleotide sequences in MUSCLE [49]. RT protein sequences used for construction of the network and the tree (Fig. 1j and Additional file 5: Figure S2, respectively) are provided in Additional file 12. The evolutionary history was inferred using the Neighbor-Joining method (Kimura-2-Parameter method) with 1000 bootstrap replicates. Positions containing missing data and gaps were removed (pairwise deletion option) leading to a total of 862 position in the final dataset. The tree was visualized as an unrooted tree. Phylogenetic network of genomic RT domain sequences from *Ty1/COPIA* LTR-retroelements was constructed using Neighbor-Net [50] within the splitstree 4.0 package [51, 52]. The phylogenetic distances were calculated by LogDet-pairwise genetic distances using LDDIST [53] with imputed missing matrix entries. Multiple sequence alignments of *CHS* genomic sequences were performed using MUSCLE [49]. The *CHS* phylogeny was inferred using the Maximum Likelihood tree based on the Kimura-2-parameter model with 1000 bootstrap replicates. *CHS* sequences were retrieved from [25]. All positions containing gaps and missing data were eliminated. There were a total of 1267 positions in the final dataset. All phylogenetic trees were constructed within MEGA 7 [54]. Phylogenetic shadowing and analysis of motif conservation was performed with mVISTA [55, 56] using LTR consensus sequences of different species prepared in BioEdit [57], allowing fasta ambiguity codes for low conserved positions. Sequences were aligned using AVID [58]. The cutoff was defined as ≥70 % conservation over a 20 bp sliding window with the minimal consensus of 7 bp relative to *A. lyrata* 5’ LTR sequence.

### Accession numbers

Short sequence reads were deposited in the NCBI GEO archive under accession number GSE69077.

## Additional files

Additional file 1: Table listing transcriptional changes of genes after 6 h HS and recovery in *A. lyrata*. (XLSX 6774 kb)
Additional file 2: Table listing transcriptional changes of genes after 6 h HS and recovery in *A. thaliana*. (XLSX 6407 kb)
Additional file 3: List of *A. lyrata* TEs in general feature format. (GFF 4554 kb)
Additional file 4: List of *A. thaliana* TEs in general feature format. (GFF 1310 kb)
Additional file 5: Figure S1. Number of TE families in *A. thaliana* (*n* = 364) and *A. lyrata* (n = 376) as identified by RepeatMasker*.* **Figure S2.** Unrooted phylogenetic tree of heat-responsive and -non-responsive *COPIA* TEs. **Table S1.** List of *ONSEN* elements in *A. thaliana* Col-0 genome. **Figure S3.** HREs found in 5’ LTRs of *ONSEN* elements. **Table S2.** List of *COPIA78*/*ONSEN* elements in *A. lyrata* MN47 genome. **Figure S4.** Percentage identity matrix of the RT nucleotide sequences of *ONSEN* elements from different species. **Figure S5.** Percentage identity matrix of LTR nucleotide sequences of *ONSEN* elements from different species. **Figure S6.** HREs found in the 5’ LTRs of *COPIA37* elements. **Figure S7.** Consensus DNA sequence of *A. lyrata TERESTRAs*. **Table S3.** List of *A. thaliana* accessions negatively tested for presence of *TERESTRA* elements. **Figure S8.** The fragment of *TERESTRA* from *A. cebennensis* clone 44. **Figure S9.** *A. halleri TERESTRA* reconstructed based on NCBI BLASTs using *A. lyrata TERESTRA* consensus sequence. **Figure S10.** HREs found in 5’ LTRs of *TERESTRA* elements. **Figure S11.** Transcriptional response of *ONSEN*, *COPIA37*, and *TERESTRA* to DNA methylation inhibitor treatments in *A. lyrata*. **Figure S12.** Density of RNA-seq reads mapping over *APUM9 – ROMANIAT5-2* region. **Figure S13.** Putative HREs in 5’/3’ LTRs of *ROMANIAT5* elements. (PDF 2864 kb)
Additional file 6: Table listing transcriptional changes of all TEs after 6 h HS and recovery in *A. lyrata*. (XLSX 6086 kb)
Additional file 7: Table with transcriptional changes of all TEs after 6 h HS and recovery in *A. thaliana*. (XLSX 1850 kb)
Additional file 8: Table showing significantly upregulated and downregulated TEs after 6 h HS and recovery in *A. lyrata*. (XLSX 63 kb)
Additional file 9: Table listing significantly upregulated and downregulated TEs after 6 h HS and recovery in *A. thaliana*. (XLSX 825 kb)
Additional file 10: Contains RT sequences of *ONSENs* from different species and was used to generate Additional file 5: Figure S4. (FASTA 15 kb)
Additional file 11: Includes LTR sequences of *ONSENs* from different species and was used to generate Additional file 5: Figure S5. (FASTA 14 kb)
Additional file 12: Contains RT amino acid sequences of *COPIA* TEs used for to generate Fig. 1j and Additional file 5: Figure S2. (FASTA 73 kb)
