# Supplementary material for: Recurrent evolution of heat-responsiveness in Brassicaceae COPIA elements
Source: Genome Biol. 2016 Oct 11;17:209. doi: 10.1186/s13059-016-1072-3 (PMC5059998; doi:10.1186/s13059-016-1072-3)
Supplement: Additional file 5: Figure S1. — Number of TE families in A. thaliana (n = 364) and A. lyrata (n = 376) as identified by RepeatMasker. Figure S2. Unrooted phylogenetic tree of heat-responsive and -non-responsive COPIA TEs. Table S1. List of ONSEN elements in A. thaliana Col-0 genome. Figure S3. HREs found in 5’ LTRs of ONSEN elements. Table S2. List of COPIA78/ONSEN elements in A. lyrata MN47 genome. Figure S4. Percentage identity matrix of the RT nucleotide sequences of ONSEN elements from different species. Figure S5. Percentage identity matrix of LTR nucleotide sequences of ONSEN elements from different species. Figure S6. HREs found in the 5’ LTRs of COPIA37 elements. Figure S7. Consensus DNA sequence of A. lyrata TERESTRAs. Table S3. List of A. thaliana accessions negatively tested for presence of TERESTRA elements. Figure S8. The fragment of TERESTRA from A. cebennensis clone 44. Figure S9. A. halleri TERESTRA reconstructed based on NCBI BLASTs using A. lyrata TERESTRA consensus sequence. Figure S10. HREs found in 5’ LTRs of TERESTRA elements. Figure S11. Transcriptional response of ONSEN, COPIA37, and TERESTRA to DNA methylation inhibitor treatments in A. lyrata. Figure S12. Density of RNA-seq reads mapping over APUM9 – ROMANIAT5-2 region. Figure S13. Putative HREs in 5’/3’ LTRs of ROMANIAT5 elements. (PDF 2864 kb) [file 13059_2016_1072_MOESM5_ESM.pdf]

### Supplemental Data – Additional file 5

Pietzenuk *et al.*, Recurrent evolution of heat-responsiveness in *Brassicaceae* COPIA elements.

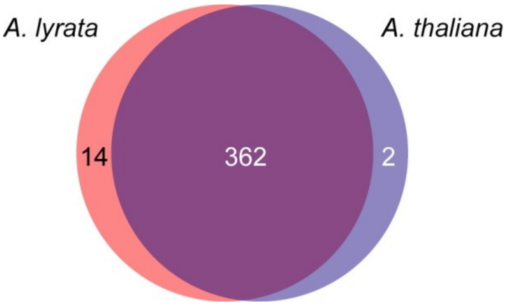


**Figure S1.** Number of transposable element families in *A. thaliana* (n = 364) and *A. lyrata* (n = 376) as identified by RepeatMasker*.* The overlap shows number of families common to both species and not overlapping parts the numbers of species-specific families.

**Figure S2.** Unrooted phylogenetic tree of heat-responsive and -non-responsive *COPIA* TEs. The tree is based on the reverse transcriptase amino acid sequences (Additional file 10) of the following heat-responsive (*ONSEN*, *TERESTRA*, *COPIA37* and *ROMANIAT5*) and -non-responsive (*COPIA19*, *COPIA23*, *COPIA35*, *COPIA45*, *COPIA66*, *COPIA75*) *COPIA* TEs.

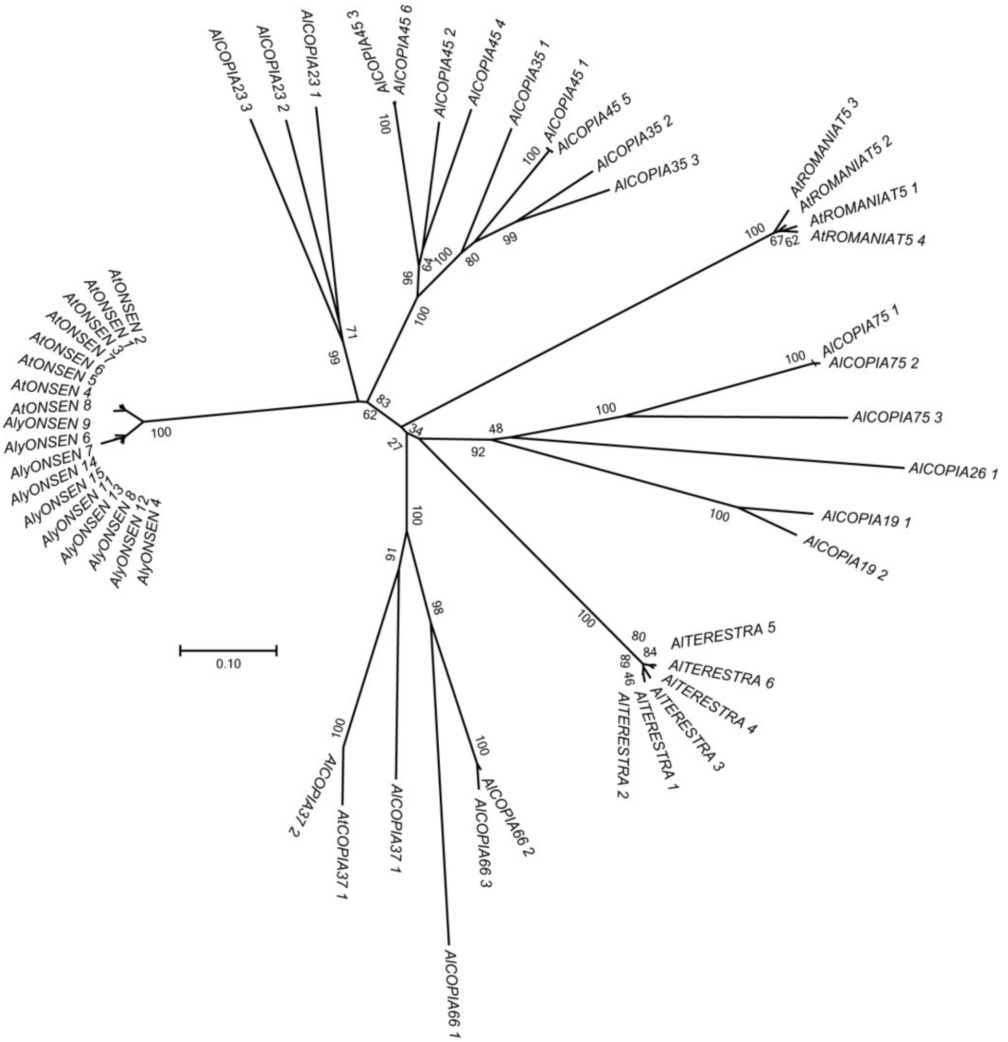

**Table S1.** List of *ONSEN* elements in *A. thaliana* Col-0 genome. *Heat responsiveness is based on the RNA-sequencing data of this study. **Names of *ONSEN1* to *ONSEN8* are based on Cavrak et al., 2014; HRE - Heat responsive element. RT - reverse transcriptase. ***Additional sequence insertion between 5' LTR and gag polyplotein domain.

| | Element** | AGI code | Chr | Start | End | Size | Type | HRE |
|---|---|---|---|---|---|---|---|---|
| Heat responsive* | *AtONSEN1* | AT1TE12295 | 1 | 3780765 | 3785720 | 4956 | full-length | 4P |
| | *AtONSEN2* | AT3TE92525 | 3 | 22695566 | 22700521 | 4956 | full-length | 4P |
| | *AtONSEN3* | AT5TE15240 | 5 | 4208083 | 4213084 | 5002 | full-length | 4P |
| | *AtONSEN4* | AT1TE71045 | 1 | 21524995 | 21529850 | 4855 | full-length | 3P |
| | *AtONSEN5* | AT1TE59755 | 1 | 18013162 | 18018117 | 5273 | full-length | 4P |
| | *AtONSEN6* | AT3TE89830 | 3 | 22059535 | 22064329 | 4795 | full-length | 4P |
| | *AtONSEN7* | AT1TE24850 | 1 | 7714708 | 7715145 | 438 | full-length*** | 4P |
| | | | | 7717356 | 7722547 | 5191 | | |
| | *AtONSEN8* | AT3TE54550 | 3 | 13369174 | 13374107 | 4934 | full-length | 4P |
| Heat-non responsive* | | | | | | | | |
| | | | | | | | | |
| | *AtONSEN9* | AT1TE57015 | 1 | 17201388 | 17201573 | 185 | truncated solo LTR | none |
| | | | | 17201917 | 17202175 | 258 | | |
| | | | | | | | | |
| | *AtONSEN10* | AT1TE10935 | 1 | 3360540 | 3361316 | 776 | pre-Integrase region | none |
| | *AtONSEN11* | AT2TE45420 | 2 | 10582288 | 10582517 | 229 | fragmented solo LTR | none |
| | *AtONSEN12* | AT2TE26505 | 2 | 6483643 | 6483961 | 318 | fragmented solo LTR | none |
| | | | | | | | | |
| | *AtONSEN13* | AT2TE12265 | 2 | 2780086 | 2781859 | 1773 | fragment - RT & Rnase_H1 | none |
| | *AtONSEN14* | AT2TE62740 | 2 | 14190558 | 14191027 | 469 | RT fragment | none |
| | *AtONSEN15* | AT3TE59075 | 3 | 14401313 | 14403971 | 2658 | RT & Rnase_H1+3´LTR | none |
| | *AtONSEN16* | AT3TE59080 | 3 | 14403653 | 14403946 | 293 | fragment of 5´LTR and gag-polyprotein | none |
| | | | | | | | | |
| | | | | | | | | |
| | *AtONSEN17* | AT3TE31680 | 3 | 7539914 | 7540288 | 274 | solo LTR | none |
| | *AtONSEN18* | AT4TE42010 | 4 | 9320200 | 9320565 | 265 | truncated solo LTR | none |

**Figure S3.** Heat responsive elements (HREs) found in 5’ long terminal repeats of *ONSEN* elements. Only heat-responsive elements are shown for *A. lyrata* and *A. thaliana*. All elements are displayed for *other species*. HRE types: red – 4P, orange – 3P, yellow – gap, green – step.

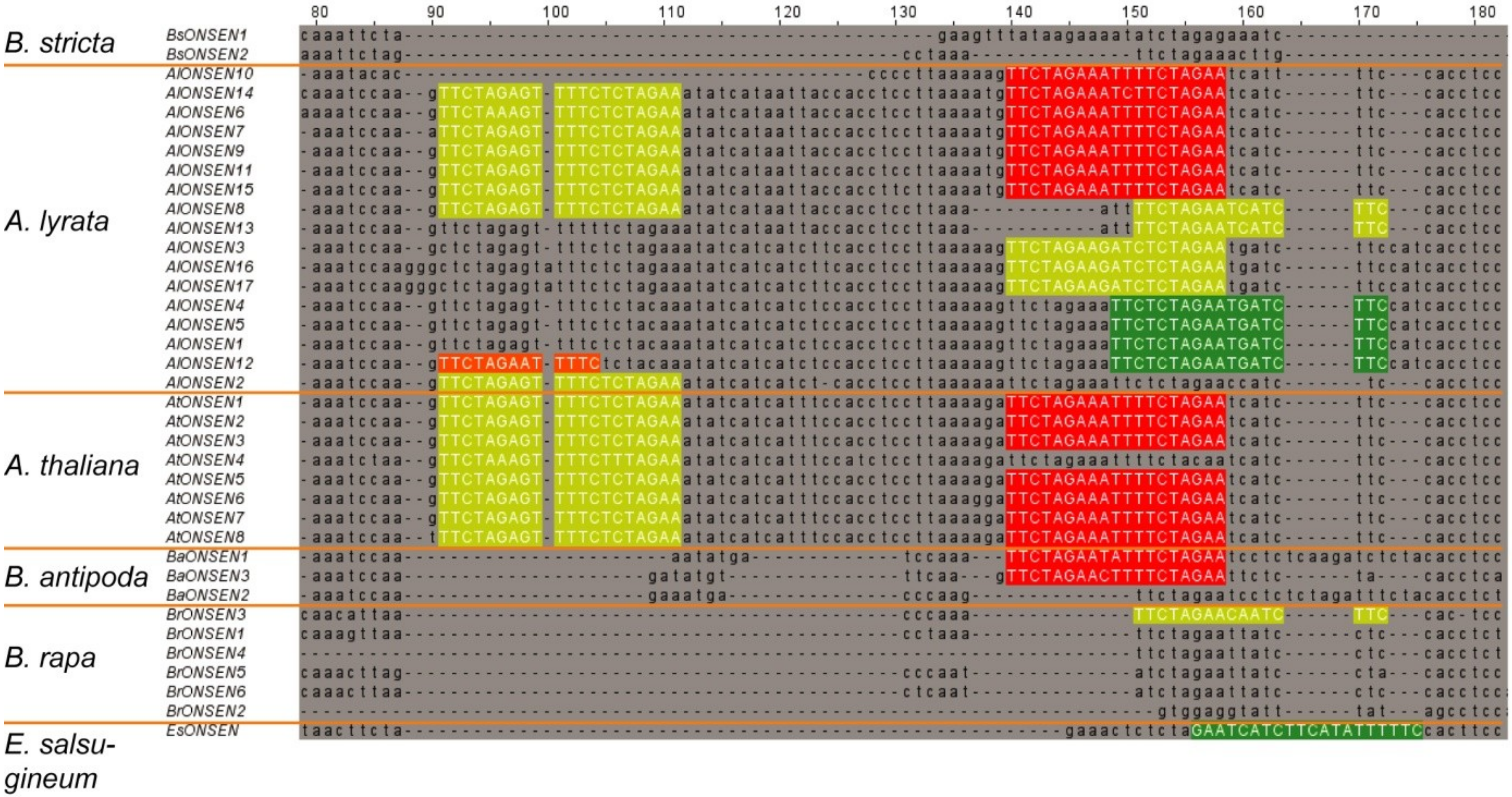

**Table S2.** List of *COPIA78*/*ONSEN* elements in *Arabidopsis lyrata* MN47 genome.
*Heat responsiveness is based on RNA-sequencing data of this study. HRE - Heat responsive element; contig end - the element is at the edge of particular genome assembly contig; n.a. - not analyzed; **Heat responsiveness based only on multiply mapping RNA-seq reads; ***Not heat responsive in RNA-sequencing, possibly due to small size. RT - reverse transcriptase.

| | Element | Scaffold | Start | End | Size | Type | HRE | Ito et al. (2013) |
|---|---|---|---|---|---|---|---|---|
| Heat responsive* | *AlONSEN1* | 1 | 11268872 | 11273881 | 5010 | full-length | gap/step | 5 |
| | *AlONSEN2* | 1 | 24919344 | 24919772 | 429 | solo LTR | gap | n.a. |
| | *AlONSEN3* | 2 | 1120762 | 1121196 | 440 | solo LTR | none** | n.a. |
| | *AlONSEN4* | 2 | 4268383 | 4273330 | 4978 | full-length | gap/step | 3 |
| | *AlONSEN5* | 2 | 12788861 | 12789817 | 956 | solo LTR | gap/step | n.a. |
| | *AlONSEN6* | 3 | 13033504 | 13038475 | 4972 | full-length | gap + 4P | 2 |
| | *AlONSEN7* | 3 | 14350695 | 14355704 | 5010 | full-length | gap + 4P | 1 |
| | *AlONSEN8* | 3 | 23055604 | 23059108 | 3504 | contig end | gap | n.a. |
| | *AlONSEN9* | 4 | 16304811 | 16309766 | 4956 | full-length | gap + 4P | 10 |
| | *AlONSEN10* | 5 | 4327023 | 4327410 | 388 | solo LTR | 4P | 17 |
| | *AlONSEN11* | 5 | 9883484 | 9888427 | 4944 | full-length | gap + 4P | 7 |
| | *AlONSEN12* | 6 | 22653438 | 22658398 | 4961 | full-length | gap | 4 |
| | *AlONSEN13* | 7 | 23781774 | 23786710 | 4937 | full-length | gap | 7 |
| | *AlONSEN14* | 8 | 15966326 | 15970217 | 3892 | full-length | gap + 4P | n.a. |
| | *AlONSEN15* | 247 | 3711 | 8654 | 4944 | full-length | gap + 4P | 8 |
| | *AlONSEN16* | 1007 | 2411 | 3818 | 1408 | contig end | none** | n.a. |
| | *AlONSEN17* | 638 | 3577 | 4818 | 1242 | contig end | gap + 4P | 12 |
| | *AlONSEN18* | 7 | 8283764 | 8284127 | 363 | fragmented solo LTR | none | n.a. |
| | *AlONSEN19* | 1007 | 42 | 1686 | 1645 | RT | none | n.a. |
| Heat-non responsive* | *AlONSEN20* | 3 | 14003080 | 14004319 | 1239 | truncated 5´LTR & gag-polypeptide | none | n.a. |
| | *AlONSEN21* | 3 | 14016614 | 14019627 | 3013 | RT & RNase H1 & 3´LTR | none | n.a. |
| | *AlONSEN22* | 1 | 10332397 | 10332510 | 113 | 3´LTR fragment | none | n.a. |
| | *AlONSEN23* | 1 | 13615947 | 13616317 | 370 | fragmented solo LTR | none | n.a. |
| | *AlONSEN24* | 1 | 24919900 | 24920073 | 173 | solo LTR fragment | none | n.a. |
| | *AlONSEN25* | 1 | 25691749 | 25692168 | 419 | RT fragment | none | n.a. |
| | *AlONSEN26* | 1 | 25692678 | 25692869 | 191 | RNase H1 fragment | none | n.a. |
| | *AlONSEN27* | 2 | 11661689 | 11661997 | 308 | solo LTR fragment | none | n.a. |
| | *AlONSEN28* | 2 | 10259067 | 10259437 | 370 | fragment | none | n.a. |
| | *AlONSEN29* | 2 | 10974958 | 10975001 | 43 | LTR fragment | none | n.a. |
| | *AlONSEN30* | 2 | 11911062 | 11911137 | 75 | LTR fragment | none | n.a. |
| | *AlONSEN31* | 3 | 9491471 | 9491721 | 250 | fragment | none | n.a. |
| | *AlONSEN32* | 3 | 14002611 | 14002810 | 199 | truncated solo LTR | 4P*** | n.a. |

| | | | | | | | | |
|---|---|---|---|---|---|---|---|---|
| | *AIONSEN33* | 3 | 23040531 | 23040611 | 80 | solo LTR fragment | none | n.a. |
| | *AIONSEN34* | 4 | 15133412 | 15133767 | 355 | solo LTR fragment | none | n.a. |
| | *AIONSEN35* | 4 | 15138010 | 15138365 | 355 | solo LTRfragment | none | n.a. |
| | *AIONSEN36* | 5 | 18806876 | 18807231 | 355 | solo LTR & fragments | none | n.a. |
| | *AIONSEN37* | 5 | 9649982 | 9650265 | 283 | solo LTR fragment | none | n.a. |
| | *AIONSEN38* | 5 | 9943036 | 9943399 | 363 | solo LTR fragment | none | n.a. |
| | *AIONSEN39* | 5 | 11358443 | 11358638 | 195 | solo LTR fragment | none | n.a. |
| | *AIONSEN40* | 5 | 12394243 | 12394562 | 319 | fragment | none | n.a. |
| | *AIONSEN41* | 5 | 12398029 | 12398350 | 321 | fragment | none | n.a. |
| | *AIONSEN42* | 5 | 12560877 | 12561493 | 616 | gag-polypeptide | none | n.a. |
| | *AIONSEN43* | 5 | 13036269 | 13036319 | 50 | solo LTR fragment | none | n.a. |
| | *AIONSEN44* | 5 | 18802278 | 18802633 | 355 | fragmented solo LTR | none | n.a. |
| | *AIONSEN45* | 7 | 3003523 | 3003877 | 354 | fragmented solo LTR | none | n.a. |
| | *AIONSEN46* | 7 | 8288370 | 8288733 | 363 | fragmented solo LTR | none | n.a. |
| | *AIONSEN47* | 7 | 14850284 | 14850721 | 437 | solo LTR | none | 16 |
| | *AIONSEN48* | 7 | 10045372 | 10046134 | 762 | fragmented solo LTR | none | n.a. |
| | *AIONSEN49* | 7 | 13857185 | 13857510 | 325 | Integrase core domain fragment | none | n.a. |
| | *AIONSEN50* | 7 | 20135759 | 20135891 | 132 | RT fragment | none | n.a. |
| | *AIONSEN51* | 7 | 23674987 | 23675327 | 340 | truncated solo LTR | none | n.a. |
| | *AIONSEN52* | 8 | 6163632 | 6163987 | 355 | fragmented solo LTR | none | n.a. |
| | *AIONSEN53* | 8 | 6159031 | 6159386 | 355 | fragmented solo LTR | none | n.a. |
| | *AIONSEN54* | 8 | 15685300 | 15685663 | 363 | fragment | none | n.a. |
| | *AIONSEN55* | 8 | 17245916 | 17246222 | 306 | fragment | none | n.a. |

**Figure S4.** Percentage identity matrix of reverse transcriptase nucleotide sequences of *ONSEN* elements from different species. LTR identity: Pink = >80%. All elements for which the full reverse transcriptase sequence was available are shown. None of the *B. antipoda* contigs contained *ONSEN* reverse transcriptase sequence and therefore it is not presented here.

| | *BrONSEN3* | *BsONSEN1* | *BsONSEN2* | *EsONSEN* | *AtONSEN8* | *AtONSEN7* | *AtONSEN5* | *AtONSEN3* | *AtONSEN6* | *AtONSEN4* | *AtONSEN1* | *AtONSEN2* | *AlONSEN7* | *AlONSEN9* | *AlONSEN6* | *AlONSEN14* | *AlONSEN4* | *AlONSEN11* | *AlONSEN15* | *AlONSEN12* | *AlONSEN8* | *AlONSEN13* |
|---|---|---|---|---|---|---|---|---|---|---|---|---|---|---|---|---|---|---|---|---|---|---|
| *BrONSEN3* | 100.0 | 84.1 | 84.6 | 84.1 | 84.3 | 84.0 | 84.4 | 84.2 | 84.2 | 84.5 | 84.5 | 84.4 | 82.5 | 85.1 | 84.4 | 85.1 | 84.4 | 84.5 | 84.5 | 84.7 | 84.8 | 84.8 |
| *BsONSEN1* | 84.1 | 100.0 | 95.1 | 85.5 | 86.6 | 86.7 | 86.9 | 86.9 | 87.0 | 87.0 | 86.7 | 86.6 | 84.2 | 86.6 | 87.0 | 87.1 | 86.5 | 86.6 | 86.6 | 86.9 | 87.0 | 87.0 |
| *BsONSEN2* | 84.6 | 95.1 | 100.0 | 85.8 | 87.0 | 86.9 | 87.0 | 87.3 | 87.1 | 87.4 | 87.1 | 87.0 | 81.7 | 86.6 | 87.0 | 87.1 | 86.5 | 86.6 | 86.6 | 86.9 | 87.0 | 87.0 |
| *EsONSEN* | 84.1 | 85.5 | 85.8 | 100.0 | 87.8 | 87.3 | 87.3 | 87.3 | 87.3 | 87.5 | 87.3 | 87.3 | 86.7 | 88.7 | 88.0 | 88.5 | 88.0 | 87.8 | 87.8 | 88.2 | 88.5 | 88.7 |
| *AtONSEN8* | 84.3 | 86.6 | 87.0 | 87.8 | 100.0 | 97.8 | 98.0 | 98.1 | 98.1 | 98.4 | 98.1 | 98.0 | 90.8 | 93.7 | 93.6 | 94.1 | 93.6 | 93.7 | 93.7 | 93.7 | 93.9 | 94.1 |
| *AtONSEN7* | 84.0 | 86.7 | 86.9 | 87.3 | 97.8 | 100.0 | 99.1 | 98.9 | 99.2 | 99.2 | 98.9 | 98.8 | 91.7 | 94.2 | 94.2 | 94.7 | 93.9 | 94.3 | 94.3 | 94.0 | 94.2 | 94.2 |
| *AtONSEN5* | 84.4 | 86.9 | 87.0 | 87.3 | 98.0 | 99.1 | 100.0 | 99.1 | 99.3 | 99.3 | 99.1 | 98.9 | 90.8 | 94.4 | 94.3 | 94.9 | 94.0 | 94.4 | 94.4 | 94.2 | 94.3 | 94.3 |
| *AtONSEN3* | 84.2 | 86.9 | 87.3 | 87.3 | 98.1 | 98.9 | 99.1 | 100.0 | 99.2 | 99.5 | 99.5 | 99.3 | 91.7 | 94.3 | 94.6 | 95.0 | 94.2 | 94.6 | 94.6 | 94.3 | 94.4 | 94.4 |
| *AtONSEN6* | 84.2 | 87.0 | 87.1 | 87.3 | 98.1 | 99.2 | 99.3 | 99.2 | 100.0 | 99.5 | 99.5 | 99.3 | 91.7 | 94.3 | 94.4 | 95.0 | 94.2 | 94.6 | 94.6 | 94.3 | 94.4 | 94.4 |
| *AtONSEN4* | 84.5 | 87.0 | 87.4 | 87.5 | 98.4 | 99.2 | 99.3 | 99.5 | 99.5 | 100.0 | 99.5 | 99.3 | 91.7 | 94.6 | 94.7 | 95.3 | 94.4 | 94.9 | 94.9 | 94.6 | 94.7 | 94.7 |
| *AtONSEN1* | 84.5 | 86.7 | 87.1 | 87.3 | 98.1 | 98.9 | 99.1 | 99.5 | 99.5 | 99.5 | 100.0 | 99.9 | 91.7 | 94.3 | 94.4 | 95.0 | 94.2 | 94.6 | 94.6 | 94.3 | 94.4 | 94.4 |
| *AtONSEN2* | 84.4 | 86.6 | 87.0 | 87.3 | 98.0 | 98.8 | 98.9 | 99.3 | 99.3 | 99.3 | 99.9 | 100.0 | 91.7 | 94.2 | 94.3 | 94.9 | 94.0 | 94.4 | 94.4 | 94.2 | 94.3 | 94.3 |
| *AlONSEN7* | 82.5 | 84.2 | 81.7 | 86.7 | 90.8 | 91.7 | 90.8 | 91.7 | 91.7 | 91.7 | 91.7 | 91.7 | 100.0 | 95.8 | 97.5 | 97.5 | 96.7 | 97.5 | 97.5 | 96.7 | 96.7 | 96.7 |
| *AlONSEN9* | 85.1 | 86.6 | 86.6 | 88.7 | 93.7 | 94.2 | 94.4 | 94.3 | 94.3 | 94.6 | 94.3 | 94.2 | 95.8 | 100.0 | 97.2 | 97.7 | 98.0 | 98.1 | 98.1 | 98.1 | 98.0 | 98.0 |
| *AlONSEN6* | 84.4 | 87.0 | 87.0 | 88.0 | 93.6 | 94.2 | 94.3 | 94.6 | 94.4 | 94.7 | 94.4 | 94.3 | 97.5 | 97.2 | 100.0 | 99.2 | 98.4 | 98.5 | 98.5 | 98.2 | 98.4 | 98.4 |
| *AlONSEN14* | 85.1 | 87.1 | 87.1 | 88.5 | 94.1 | 94.7 | 94.9 | 95.0 | 95.0 | 95.3 | 95.0 | 94.9 | 97.5 | 97.7 | 99.2 | 100.0 | 98.6 | 99.1 | 99.1 | 98.8 | 98.9 | 98.9 |
| *AlONSEN4* | 84.4 | 86.5 | 86.5 | 88.0 | 93.6 | 93.9 | 94.0 | 94.2 | 94.2 | 94.4 | 94.2 | 94.0 | 96.7 | 98.0 | 98.4 | 98.6 | 100.0 | 99.1 | 99.1 | 99.1 | 98.9 | 98.9 |
| *AlONSEN11* | 84.5 | 86.6 | 86.6 | 87.8 | 93.7 | 94.3 | 94.4 | 94.6 | 94.6 | 94.9 | 94.6 | 94.4 | 97.5 | 98.1 | 98.5 | 99.1 | 99.1 | 100.0 | 100.0 | 99.2 | 99.1 | 99.1 |
| *AlONSEN15* | 84.5 | 86.6 | 86.6 | 87.8 | 93.7 | 94.3 | 94.4 | 94.6 | 94.6 | 94.9 | 94.6 | 94.4 | 97.5 | 98.1 | 98.5 | 99.1 | 99.1 | 100.0 | 100.0 | 99.2 | 99.1 | 99.1 |
| *AlONSEN12* | 84.7 | 86.9 | 86.9 | 88.2 | 93.7 | 94.0 | 94.2 | 94.3 | 94.3 | 94.6 | 94.3 | 94.2 | 96.7 | 98.1 | 98.2 | 98.8 | 99.1 | 99.2 | 99.2 | 100.0 | 99.1 | 99.1 |
| *AlONSEN8* | 84.8 | 87.0 | 87.0 | 88.5 | 93.9 | 94.2 | 94.3 | 94.4 | 94.4 | 94.7 | 94.4 | 94.3 | 96.7 | 98.0 | 98.4 | 98.9 | 98.9 | 99.1 | 99.1 | 99.1 | 100.0 | 99.7 |
| *AlONSEN13* | 84.8 | 87.0 | 87.0 | 88.7 | 94.1 | 94.2 | 94.3 | 94.4 | 94.4 | 94.7 | 94.4 | 94.3 | 96.7 | 98.0 | 98.4 | 98.9 | 98.9 | 99.1 | 99.1 | 99.1 | 99.7 | 100.0 |

**Figure S5.** Percentage identity matrix of LTR nucleotide sequences of *ONSEN* elements from different species. LTR identity: Pink = >80%, yellow = 70-80%, green = 60-70%, no color = <60%. LTRs were clustered according to their similarity.

| | *BrONSEN2* | *BsONSEN1* | *BsONSEN2* | *EsONSEN* | *AlONSEN2* | *BaONSEN1* | *BaONSEN2* | *BaONSEN3* | *AtONSEN4* | *AtONSEN8* | *AtONSEN7* | *AtONSEN6* | *AtONSEN5* | *AtONSEN2* | *AtONSEN1* | *AtONSEN3* | *AlONSEN10* | *AlONSEN14* | *AlONSEN6* | *AlONSEN7* | *AlONSEN9* | *AlONSEN11* | *AlONSEN15* | *AlONSEN8* | *AlONSEN13* | *AlONSEN3* | *AlONSEN16* | *AlONSEN17* | *AlONSEN4* | *AlONSEN5* | *AlONSEN1* | *AlONSEN12* | *BrONSEN3* | *BrONSEN1* | *BrONSEN4* | *BrONSEN05* | *BrONSEN6* |
|---|---|---|---|---|---|---|---|---|---|---|---|---|---|---|---|---|---|---|---|---|---|---|---|---|---|---|---|---|---|---|---|---|---|---|---|---|---|
| *BrONSEN2* | 100.0 | 60.8 | 64.3 | 64.9 | 60.1 | 64.1 | 61.5 | 61.4 | 63.5 | 62.4 | 62.3 | 62.4 | 62.4 | 62.4 | 62.4 | 62.0 | 64.5 | 64.7 | 64.7 | 62.4 | 63.5 | 64.1 | 63.5 | 64.1 | 64.7 | 65.9 | 67.5 | 67.5 | 64.7 | 64.7 | 65.9 | 65.9 | 63.6 | 61.5 | 64.1 | 59.6 | 62.3 |
| *BsONSEN1* | 60.8 | 100.0 | 84.1 | 57.3 | 59.9 | 67.5 | 63.7 | 63.8 | 68.0 | 66.4 | 67.1 | 68.3 | 66.9 | 67.5 | 67.5 | 67.5 | 68.0 | 69.4 | 67.7 | 69.0 | 69.0 | 68.4 | 68.4 | 68.3 | 68.6 | 68.1 | 65.7 | 65.7 | 67.4 | 67.4 | 67.6 | 68.1 | 63.3 | 65.1 | 75.7 | 66.9 | 67.7 |
| *BsONSEN2* | 64.3 | 84.1 | 100.0 | 61.5 | 64.6 | 72.7 | 70.0 | 70.1 | 74.5 | 72.7 | 73.3 | 73.9 | 73.0 | 73.6 | 73.6 | 73.6 | 73.9 | 74.1 | 75.7 | 76.0 | 75.7 | 76.0 | 75.7 | 75.1 | 75.1 | 75.7 | 73.0 | 73.0 | 74.5 | 74.5 | 73.9 | 73.6 | 71.6 | 69.9 | 76.6 | 73.9 | 70.6 |
| *EsONSEN* | 64.9 | 57.3 | 61.5 | 100.0 | 66.8 | 73.7 | 72.1 | 69.9 | 72.4 | 72.7 | 72.4 | 73.3 | 73.0 | 73.0 | 73.0 | 73.1 | 73.3 | 72.9 | 73.2 | 74.0 | 74.7 | 74.3 | 73.7 | 73.5 | 74.1 | 73.6 | 72.5 | 72.5 | 75.2 | 75.2 | 73.7 | 73.7 | 69.7 | 67.5 | 78.6 | 67.3 | 69.3 |
| *AlONSEN2* | 60.1 | 59.9 | 64.6 | 66.8 | 100.0 | 65.7 | 66.9 | 77.2 | 75.8 | 75.4 | 76.0 | 76.5 | 76.5 | 76.7 | 76.7 | 76.8 | 74.3 | 74.9 | 78.2 | 79.0 | 79.0 | 78.8 | 78.5 | 78.4 | 78.7 | 80.6 | 77.9 | 77.9 | 81.9 | 81.9 | 80.3 | 80.3 | 66.1 | 64.7 | 76.5 | 67.5 | 65.1 |
| *BaONSEN1* | 64.1 | 67.5 | 72.7 | 73.7 | 65.7 | 100.0 | 79.2 | 82.8 | 76.5 | 76.5 | 76.8 | 76.5 | 76.2 | 76.8 | 76.8 | 76.5 | 75.9 | 77.0 | 78.0 | 78.6 | 78.6 | 79.3 | 78.6 | 77.1 | 77.8 | 78.5 | 78.4 | 78.4 | 77.5 | 77.5 | 77.0 | 77.0 | 71.8 | 74.3 | 85.4 | 72.4 | 74.8 |
| *BaONSEN2* | 61.5 | 63.7 | 70.0 | 72.1 | 66.9 | 79.2 | 100.0 | 82.0 | 74.7 | 73.1 | 73.4 | 74.7 | 73.4 | 74.0 | 74.0 | 74.0 | 73.2 | 76.5 | 75.1 | 77.9 | 75.7 | 76.6 | 76.6 | 75.7 | 76.3 | 77.2 | 77.6 | 77.6 | 74.9 | 74.9 | 75.6 | 75.6 | 71.1 | 70.7 | 79.5 | 68.2 | 70.8 |
| *BaONSEN3* | 61.4 | 63.8 | 70.1 | 69.9 | 77.2 | 82.8 | 82.0 | 100.0 | 78.7 | 77.2 | 79.4 | 79.4 | 78.7 | 79.4 | 79.4 | 79.4 | 78.3 | 80.0 | 81.8 | 81.8 | 81.0 | 82.5 | 81.8 | 81.3 | 81.3 | 81.0 | 81.9 | 81.9 | 78.8 | 78.8 | 79.6 | 78.8 | 69.0 | 73.9 | 82.2 | 72.2 | 75.9 |
| *AtONSEN4* | 63.5 | 68.0 | 74.5 | 72.4 | 75.8 | 76.5 | 74.7 | 78.7 | 100.0 | 95.3 | 95.8 | 96.5 | 96.3 | 96.8 | 96.8 | 96.8 | 86.6 | 90.3 | 89.1 | 90.1 | 89.9 | 90.4 | 90.1 | 89.6 | 89.3 | 88.6 | 88.3 | 88.3 | 86.2 | 86.2 | 87.1 | 86.6 | 72.3 | 73.3 | 83.8 | 73.1 | 76.0 |
| *AtONSEN8* | 62.4 | 66.4 | 72.7 | 72.7 | 75.4 | 76.5 | 73.1 | 77.2 | 95.3 | 100.0 | 97.7 | 98.0 | 98.2 | 98.2 | 98.2 | 98.2 | 88.6 | 92.9 | 91.1 | 92.3 | 91.8 | 92.7 | 92.5 | 92.1 | 91.8 | 89.0 | 90.0 | 90.0 | 88.8 | 88.8 | 89.5 | 89.1 | 71.0 | 71.1 | 83.8 | 73.1 | 75.7 |
| *AtONSEN7* | 62.3 | 67.1 | 73.3 | 72.4 | 76.0 | 76.8 | 73.4 | 79.4 | 95.8 | 97.7 | 100.0 | 98.4 | 98.6 | 99.1 | 99.1 | 99.1 | 88.2 | 92.0 | 91.5 | 92.4 | 92.6 | 92.6 | 92.4 | 92.2 | 92.0 | 88.9 | 89.4 | 89.4 | 88.7 | 88.7 | 89.2 | 88.7 | 71.0 | 71.4 | 83.1 | 72.8 | 76.0 |
| *AtONSEN6* | 62.4 | 68.3 | 73.9 | 73.3 | 76.5 | 76.5 | 74.7 | 79.4 | 96.5 | 98.0 | 98.4 | 100.0 | 98.9 | 99.3 | 99.3 | 99.3 | 88.1 | 92.6 | 91.3 | 92.7 | 92.5 | 92.9 | 92.7 | 92.5 | 92.3 | 89.2 | 89.7 | 89.7 | 88.8 | 88.8 | 89.5 | 89.1 | 71.0 | 71.4 | 83.1 | 73.4 | 76.0 |
| *AtONSEN5* | 62.4 | 66.9 | 73.0 | 73.0 | 76.5 | 76.2 | 73.4 | 78.7 | 96.3 | 98.2 | 98.6 | 98.9 | 100.0 | 99.6 | 99.6 | 99.6 | 88.1 | 92.9 | 91.3 | 92.5 | 92.3 | 92.7 | 92.5 | 92.1 | 91.8 | 89.0 | 89.4 | 89.4 | 88.5 | 88.5 | 89.3 | 88.8 | 71.0 | 71.1 | 83.1 | 72.8 | 75.4 |
| *AtONSEN2* | 62.4 | 67.5 | 73.6 | 73.0 | 76.7 | 76.8 | 74.0 | 79.4 | 96.8 | 98.2 | 99.1 | 99.3 | 99.6 | 100.0 | 100.0 | 100.0 | 88.6 | 92.6 | 91.8 | 92.9 | 92.7 | 93.2 | 92.9 | 92.5 | 92.3 | 89.4 | 90.0 | 90.0 | 89.0 | 89.0 | 89.8 | 89.3 | 71.3 | 71.6 | 83.8 | 73.4 | 76.0 |
| *AtONSEN1* | 62.4 | 67.5 | 73.6 | 73.0 | 76.7 | 76.8 | 74.0 | 79.4 | 96.8 | 98.2 | 99.1 | 99.3 | 99.6 | 100.0 | 100.0 | 100.0 | 88.6 | 92.6 | 91.8 | 92.9 | 92.7 | 93.2 | 92.9 | 92.5 | 92.3 | 89.4 | 90.0 | 90.0 | 89.0 | 89.0 | 89.8 | 89.3 | 71.3 | 71.6 | 83.8 | 73.4 | 76.0 |
| *AtONSEN3* | 62.0 | 67.5 | 73.6 | 73.1 | 76.8 | 76.5 | 74.0 | 79.4 | 96.8 | 98.2 | 99.1 | 99.3 | 99.6 | 100.0 | 100.0 | 100.0 | 88.6 | 92.6 | 91.8 | 92.9 | 92.7 | 93.2 | 92.9 | 92.5 | 92.3 | 89.4 | 90.0 | 90.0 | 89.0 | 89.0 | 89.8 | 89.3 | 71.3 | 71.6 | 83.8 | 73.4 | 76.0 |
| *AlONSEN10* | 64.5 | 68.0 | 73.9 | 73.3 | 74.3 | 75.9 | 73.2 | 78.3 | 86.6 | 88.6 | 88.2 | 88.1 | 88.1 | 88.6 | 88.6 | 88.6 | 100.0 | 91.8 | 93.3 | 92.3 | 92.8 | 93.0 | 92.3 | 93.1 | 93.1 | 90.1 | 90.0 | 90.0 | 90.0 | 90.0 | 89.9 | 89.9 | 72.4 | 74.3 | 85.5 | 73.1 | 74.2 |
| *AlONSEN14* | 64.7 | 69.4 | 74.1 | 72.9 | 74.9 | 77.0 | 76.5 | 80.0 | 90.3 | 92.9 | 92.0 | 92.6 | 92.9 | 92.6 | 92.6 | 92.6 | 91.8 | 100.0 | 94.9 | 96.4 | 96.5 | 97.5 | 97.0 | 96.3 | 96.1 | 91.0 | 92.5 | 92.5 | 92.2 | 92.2 | 92.9 | 92.4 | 73.8 | 74.6 | 85.6 | 75.4 | 77.4 |
| *AlONSEN6* | 64.7 | 67.7 | 75.7 | 73.2 | 78.2 | 78.0 | 75.1 | 81.8 | 89.1 | 91.1 | 91.5 | 91.3 | 91.3 | 91.8 | 91.8 | 91.8 | 93.3 | 94.9 | 100.0 | 96.2 | 96.4 | 97.3 | 96.6 | 97.7 | 97.5 | 90.9 | 91.8 | 91.8 | 93.6 | 93.6 | 93.0 | 92.7 | 72.4 | 72.7 | 85.6 | 73.6 | 75.2 |
| *AlONSEN7* | 62.4 | 69.0 | 76.0 | 74.0 | 79.0 | 78.6 | 77.9 | 81.8 | 90.1 | 92.3 | 92.4 | 92.7 | 92.5 | 92.9 | 92.9 | 92.9 | 92.3 | 96.4 | 96.2 | 100.0 | 97.7 | 98.2 | 98.0 | 97.2 | 97.0 | 91.5 | 92.6 | 92.6 | 92.9 | 92.9 | 92.7 | 92.5 | 72.8 | 73.5 | 84.4 | 75.0 | 77.0 |
| *AlONSEN9* | 63.5 | 69.0 | 75.7 | 74.7 | 79.0 | 78.6 | 75.7 | 81.0 | 89.9 | 91.8 | 92.6 | 92.5 | 92.3 | 92.7 | 92.7 | 92.7 | 92.8 | 96.5 | 96.4 | 97.7 | 100.0 | 98.2 | 97.5 | 97.5 | 97.2 | 91.5 | 92.6 | 92.6 | 92.9 | 92.9 | 92.7 | 92.5 | 73.4 | 74.1 | 86.3 | 74.4 | 76.2 |
| *AlONSEN11* | 64.1 | 68.4 | 76.0 | 74.3 | 78.8 | 79.3 | 76.6 | 82.5 | 90.4 | 92.7 | 92.6 | 92.9 | 92.7 | 93.2 | 93.2 | 93.2 | 93.0 | 97.5 | 97.3 | 98.2 | 98.2 | 100.0 | 99.3 | 98.6 | 98.4 | 92.5 | 93.7 | 93.7 | 94.1 | 94.1 | 94.1 | 93.9 | 74.0 | 74.4 | 85.6 | 75.3 | 77.3 |
| *AlONSEN15* | 63.5 | 68.4 | 75.7 | 73.7 | 78.5 | 78.6 | 76.6 | 81.8 | 90.1 | 92.5 | 92.4 | 92.7 | 92.5 | 92.9 | 92.9 | 92.9 | 92.3 | 97.0 | 96.6 | 98.0 | 97.5 | 99.3 | 100.0 | 97.9 | 97.7 | 91.8 | 92.9 | 92.9 | 93.4 | 93.4 | 93.4 | 93.2 | 73.7 | 74.1 | 85.0 | 75.0 | 76.8 |
| *AlONSEN8* | 64.1 | 68.3 | 75.1 | 73.5 | 78.4 | 77.1 | 75.7 | 81.3 | 89.6 | 92.1 | 92.2 | 92.5 | 92.1 | 92.5 | 92.5 | 92.5 | 93.1 | 96.3 | 97.7 | 97.2 | 97.5 | 98.6 | 97.9 | 100.0 | 99.3 | 91.6 | 92.7 | 92.7 | 94.2 | 94.2 | 92.8 | 92.6 | 73.4 | 73.8 | 85.6 | 75.0 | 76.0 |
| *AlONSEN13* | 64.7 | 68.6 | 75.1 | 74.1 | 78.7 | 77.8 | 76.3 | 81.3 | 89.3 | 91.8 | 92.0 | 92.3 | 91.8 | 92.3 | 92.3 | 92.3 | 93.1 | 96.1 | 97.5 | 97.0 | 97.2 | 98.4 | 97.7 | 99.3 | 100.0 | 91.8 | 93.0 | 93.0 | 94.2 | 94.2 | 93.0 | 92.8 | 73.4 | 73.8 | 86.3 | 74.4 | 76.0 |
| *AlONSEN3* | 65.9 | 68.1 | 75.7 | 73.6 | 80.6 | 78.5 | 77.2 | 81.0 | 88.6 | 89.0 | 88.9 | 89.2 | 89.0 | 89.4 | 89.4 | 89.4 | 90.1 | 91.0 | 90.9 | 91.5 | 91.5 | 92.5 | 91.8 | 91.6 | 91.8 | 100.0 | 98.2 | 98.2 | 93.8 | 93.8 | 94.6 | 94.3 | 72.5 | 72.1 | 84.4 | 72.9 | 74.0 |
| *AlONSEN16* | 67.5 | 65.7 | 73.0 | 72.5 | 77.9 | 78.4 | 77.6 | 81.9 | 88.3 | 90.0 | 89.4 | 89.7 | 89.4 | 90.0 | 90.0 | 90.0 | 90.0 | 92.5 | 91.8 | 92.6 | 92.6 | 93.7 | 92.9 | 92.7 | 93.0 | 98.2 | 100.0 | 100.0 | 95.9 | 95.9 | 96.6 | 96.1 | 73.8 | 73.0 | 85.0 | 72.7 | 75.0 |
| *AlONSEN17* | 67.5 | 65.7 | 73.0 | 72.5 | 77.9 | 78.4 | 77.6 | 81.9 | 88.3 | 90.0 | 89.4 | 89.7 | 89.4 | 90.0 | 90.0 | 90.0 | 90.0 | 92.5 | 91.8 | 92.6 | 92.6 | 93.7 | 92.9 | 92.7 | 93.0 | 98.2 | 100.0 | 100.0 | 95.9 | 95.9 | 96.6 | 96.1 | 73.8 | 73.0 | 85.0 | 72.7 | 75.0 |
| *AlONSEN4* | 64.7 | 67.4 | 74.5 | 75.2 | 81.9 | 77.5 | 74.9 | 78.8 | 86.2 | 88.8 | 88.7 | 88.8 | 88.5 | 89.0 | 89.0 | 89.0 | 90.0 | 92.2 | 93.6 | 92.9 | 92.9 | 94.1 | 93.4 | 94.2 | 94.2 | 93.8 | 95.9 | 95.9 | 100.0 | 100.0 | 97.4 | 97.2 | 74.4 | 73.9 | 83.8 | 74.0 | 76.0 |
| *AlONSEN5* | 64.7 | 67.4 | 74.5 | 75.2 | 81.9 | 77.5 | 74.9 | 78.8 | 86.2 | 88.8 | 88.7 | 88.8 | 88.5 | 89.0 | 89.0 | 89.0 | 90.0 | 92.2 | 93.6 | 92.9 | 92.9 | 94.1 | 93.4 | 94.2 | 94.2 | 93.8 | 95.9 | 95.9 | 100.0 | 100.0 | 97.4 | 97.2 | 74.4 | 73.9 | 83.8 | 74.0 | 76.0 |
| *AlONSEN1* | 65.9 | 67.6 | 73.9 | 73.7 | 80.3 | 77.0 | 75.6 | 79.6 | 87.1 | 89.5 | 89.2 | 89.5 | 89.3 | 89.8 | 89.8 | 89.8 | 89.9 | 92.9 | 93.0 | 92.7 | 92.7 | 94.1 | 93.4 | 92.8 | 93.0 | 94.6 | 96.6 | 96.6 | 97.4 | 97.4 | 100.0 | 99.3 | 72.8 | 72.9 | 83.1 | 72.4 | 74.6 |
| *AlONSEN12* | 65.9 | 68.1 | 73.6 | 73.7 | 80.3 | 77.0 | 75.6 | 78.8 | 86.6 | 89.1 | 88.7 | 89.1 | 88.8 | 89.3 | 89.3 | 89.3 | 89.9 | 92.4 | 92.7 | 92.5 | 92.5 | 93.9 | 93.2 | 92.6 | 92.8 | 94.3 | 96.1 | 96.1 | 97.2 | 97.2 | 99.3 | 100.0 | 72.8 | 72.9 | 82.5 | 72.4 | 74.6 |
| *BrONSEN3* | 63.6 | 63.3 | 71.6 | 69.7 | 66.1 | 71.8 | 71.1 | 69.0 | 72.3 | 71.0 | 71.0 | 71.0 | 71.0 | 71.3 | 71.3 | 71.3 | 72.4 | 73.8 | 72.4 | 72.8 | 73.4 | 74.0 | 73.7 | 73.4 | 73.4 | 72.5 | 73.8 | 73.8 | 74.4 | 74.4 | 72.8 | 72.8 | 100.0 | 81.4 | 84.8 | 79.4 | 75.3 |
| *BrONSEN1* | 61.5 | 65.1 | 69.9 | 67.5 | 64.7 | 74.3 | 70.7 | 73.9 | 73.3 | 71.1 | 71.4 | 71.4 | 71.1 | 71.6 | 71.6 | 71.6 | 74.3 | 74.6 | 72.7 | 73.5 | 74.1 | 74.4 | 74.1 | 73.8 | 73.8 | 72.1 | 73.0 | 73.0 | 73.9 | 73.9 | 72.9 | 72.9 | 81.4 | 100.0 | 100.0 | 83.8 | 81.0 |
| *BrONSEN4* | 64.1 | 75.7 | 76.6 | 78.6 | 76.5 | 85.4 | 79.5 | 82.2 | 83.8 | 83.8 | 83.1 | 83.1 | 83.1 | 83.8 | 83.8 | 83.8 | 85.5 | 85.6 | 85.6 | 84.4 | 86.3 | 85.6 | 85.0 | 85.6 | 86.3 | 84.4 | 85.0 | 85.0 | 83.8 | 83.8 | 83.1 | 82.5 | 84.8 | 100.0 | 100.0 | 86.9 | 88.7 |
| *BrONSEN05* | 59.6 | 66.9 | 73.9 | 67.3 | 67.5 | 72.4 | 68.2 | 72.2 | 73.1 | 73.1 | 72.8 | 73.4 | 72.8 | 73.4 | 73.4 | 73.4 | 73.1 | 75.4 | 73.6 | 75.0 | 74.4 | 75.3 | 75.0 | 75.0 | 74.4 | 72.9 | 72.7 | 72.7 | 74.0 | 74.0 | 72.4 | 72.4 | 79.4 | 83.8 | 86.9 | 100.0 | 86.8 |
| *BrONSEN6* | 62.3 | 67.7 | 70.6 | 69.3 | 65.1 | 74.8 | 70.8 | 75.9 | 76.0 | 75.7 | 76.0 | 76.0 | 75.4 | 76.0 | 76.0 | 76.0 | 74.2 | 77.4 | 75.2 | 77.0 | 76.2 | 77.3 | 76.8 | 76.0 | 76.0 | 74.0 | 75.0 | 75.0 | 76.0 | 76.0 | 74.6 | 74.6 | 75.3 | 81.0 | 88.7 | 86.8 | 100.0 |

**Figure S6.** Heat responsive elements (HREs) found in the 5' long terminal repeats of *COPIA37* elements. Only heat-responsive elements are shown for *A. lyrata* and *A. thaliana*. All elements are displayed for *C. rubella* and *E. salsugineum*. HRE types: orange – 3P, yellow – gap, green – step.

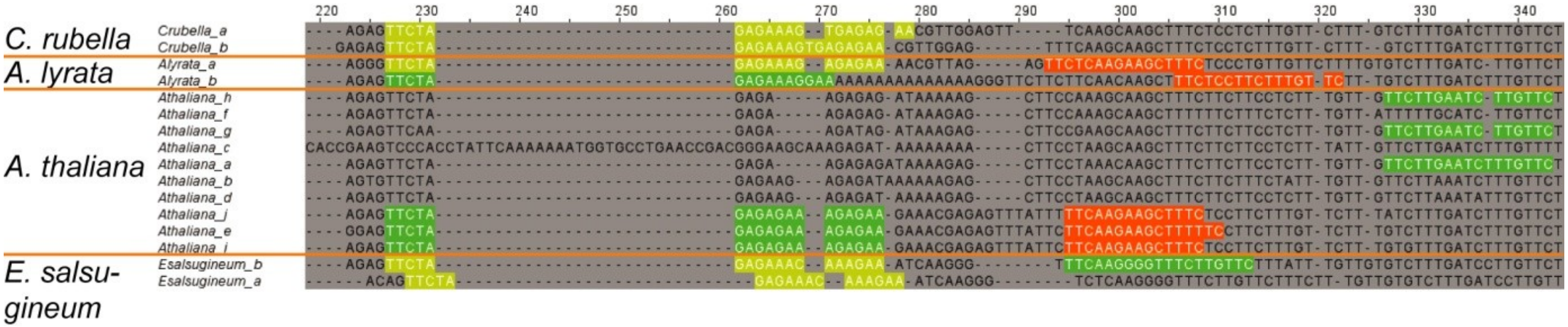

**Figure S7.** Consensus DNA sequence of *A. lyrata TERESTRAs*. Specific regions were indicated in different colors according to Fig. 3c: 5' LTR (grey), PBS (underlined), GAG polyprotein (green), integrase (violet), reverse transcriptase (yellow), RNAse H1 (blue), PPT (red), 3' LTR (grey). CG dinucleotides within 5' LTR were underlined and bold.

TGAAGTTAAACTTGATTTTGGTTTAAGCTTAATTATGGGTTTATAATTCAATATTCTATATTGGATAAAACCCAAAGTTAAGCC
TAAGTTCTAGATTTCTCTAGAGCATCATAGAAAAATCATCACCTCCTTAAATGTTCTAGAGAATTYTATTAGATAGATATTTAG
AWAATTATGTAGAATAATCTAGAAGTTTGTAAGATAAAATCTAGATATTTTAGTAGAGTTATAGAGATGATGGTAGAATAATCT
AGAGACTTTGTAAGCTAACCTTGGAGGCTATAAATACCTCCTCACCCCCMTCATTTGTAATTWTGTGAGGAAGTTGAACAAG
TGAATAAAAAGAAAGTTCAAATAAAAGAACACTCTTTAAAAGTTCTCTAAGTCTCTCCAAAAGTCTTTCAAGTAGTCTCTAATT
GTTCTTCCAAATTTTCTATCATACTTAGAGGGTTTCTTATCTTGATTATAT**CG**AGTTTCT**CG**GGTATTA**CG**GTCTTGGGCTAGT
GCTAAGCACTAT**CG**AGTT**CG**GTTTCTTTACATGGTATCACTCGAGGAGCCATTCTAATCTTGTGCTCTTCAAGATCGGGATT
ATAGGAGAACTCGGATCTACTCTAAGTATGGAATCTATGGGTCGTGTTGTTGGATTGGGAATGGAGATCTTAAACCAATCTA
ACTACCGGTTATGGAAGTCATGTATGGAGTCATACTTGGTTAGTGAGGACTTATGGGATGTCGTCGGCGGAAGTAATACTT
CACCACCTACCGGAGATGCTGCAACCGCGGAAGCGACAAAGGAGTGGACACGTAAAAATGCCAAGGCGGARTTTRCTTTA
AAGAGGTCAATATCCTCAGGAGTATTTGAGCATGTCTCAAGGTGCACATCCGCTAGTTCAATTTGGCAAGCGTTAGATCGAC
TGTTCAACAAGAARAAYGAAGCTCGTCTACAATTGTTAGAAAACGAGTTGGCRAATGCGAAGCAGGGGGAGTCTTCAATCT
CGGAGTTTTTCATCAAGGTAAAGAACCTTTGCTCTGAGATMAATAGTCTTAATCCGGAGGAATCAATCTCGGATGCTCGGTT
GAAGCGGTTTATTATTCGAGGTCTACGACCCGAGTATACACCGTTTGTGACTTCGGTTCAAGGATGGGCTACACAACCTTC
CTTGGARGAGTTTGAAAATTTGCTYGCTTCGCAAGAATCACTAGCRGTACAAATGGCGGGAGTCAAAATTCATGATGACTCG
GGRAGCGCYTTTGTAGCTAGGAGGCAACAMAACTTYAAAGCCAAATCYAATGAYGGTGGTCCAAGAAATAATGATGGACAT
GAAGGATCTTCTAAGGGAGATAAGAAAAAATTCAAGTGCTATCGGTGTGGAAAACTWGGGCATTTTAAAAARGATTGCCGA
GTCAAGCTGAAGGAAACAAACATGGCRGAATCAAAAGGTCATAATGAAGACGAAGAATGGGGAAAATGTTTTACGRTGGAG
GCTGCATCTTCAAGTACATCTACACCTAAGAARGTGGAAAATAATTGGATCGTAGATTCTGGTTGTAGCCATCATATYACYG
GAGACGAGAAATTATTTTCTAGTCTTCAACGCCATGAAGGAAAAGAAGCAATCATTACCGCGGATAATTCAATCCATCACGT
TGAGAAGGAAGGAACGGTGGTCATCAAAGGAGATGATGGAAGCCCAATCACTCTYRAGAACGTTTACCATGTTCCTGGAGT
AAAAAAGAATCTTCTCTCCGTGGTAAATGCGGTGGACTCAGGTAACTATGTYTTATTTGGCCCAAGAGATGTTAAATTCTTAA
AGAATATTCAAGAGTTAAAGGCGGAYGTGGTTCACACTGGAGCACGRGTTAAAGATCTRTACGTTTTATCGGCGTCAAACTC
TTATGTGGAGAAGATGAGTACCAACGATAATGCTTTTATATGGCATGCTAGGCTTGGTCAYATAAATATGACTAARCTCAAG
GTTATGGTRAATAAAGAYTTGGTKAACGGGCTCCCAAAGTTGAAGATCCAAGATGAAGGCACACTTTGTGAAGGATGTCAAT
ATGGAAAGTCTCATAGACTTCCATTTGACTATTCCACTTCAAGGTGCAGTGTTCCGTTGGARAGGGTCCAYAGTGACTTGAT
GGGTCCGACAAGAACACCCTCATATTYCGGGTATCRCTACATGTTGTTGTTCGTTGAYGACTTCTCWCGGTACACTTGGGT
ATACTTTGTGAAGGAAAAATCTGAAGTATTCTCCAAGTTTCTAGAATTCAAGGTTACCGTGGAAGGGGAGTTAGGTCAGAAG
ATCAAGACCTTAAGGACGGATAATGGAGGGGAGTTCATGTCTAAGGAGTTTCTCTCTTTTTGTAGAGATAATGGTATCAAGA
GAGAATTTACATGTCCGCATACACCTCAACAAAATGGAGTAGCAGAGAGGAAGATCAGACATCTAAGTGAGACGTGTAGAA
GTTGGCTCCATGGHAAAGATTTGCCTAAGGAGTTATGGGCGGAGGGMATGAGATGTGCAGCCTATGTCATCAACCGGATG
CCACTTAGTCCAAACAATATGAAGTCTCCTTATGAGATGGTTCATGGGAAGAAGCCAACGGTGAAACATCTCAGAATATTTG
GATCAGTCTGCTACGTYCATGTGTTTGATTCACAAAGAACTAAGTTGGAGGCAAAGGCGAAGAAATGCATATTTGTCGGCTA
TGATGAACAAAGGAAGGGTTGGAGGTGTATGGATCCTGAGACACACAAGTATGTTGTGTCTCGTGATGTTGTCTTTGATGAA
GTTTCTTCATACTACGGGCCACCTCAAGTCTTGGTTGAAAAAGATGGTGCRAGTTCCTCCAAGATCGATGAATCAACTCTCC
AAGTACCATGTGAGARTGGAAGTCCTGGAAATAAAATTCAAGGTCAAAGGGGGAGCACTAACCAAGAAGAAGAGGAACAAG
ATCATGGTTCGATGGTCAATCAACGGCCGAAAAGAAACATTGTCAAGCCTGCAAGATATAGAGATGAAGAATTCATCACTAC
TTATTCATGCTTCTTCGCAGCTCCGTTAGATGATGATGAGCCATCTTCTTATGATGAAGCTAAAGGAGTCCAAGAATGGGAA
GCTGCAATGAAKGAGGAGATGAGTGCTCTMAAGAAAAATGAAACTTGGGATCTGGTTCCAAAACCCAAGGATGTTGAGCCC
GTTTCTTGCAAATGGGTGTATCGAATTAAGCGAAAAGCAGATGGGAGCATTGATAGRTTTAAAGCAAGGTTGGTTGCTCGG
GGATTCTCTCAAAARTATGGAGAAGATTATGATGAGACATTYAGTCCWGTGGCGAAGATGACTACGGTACGCTCACTYTTAT
CCTTAGCAGCAWGYTTTGGTTGGAAGTTATGGCAACTTGATGTGAAGAATGCCTTTCTATATGGTGAGCTTGATAAAAGTAT
CTTCATGGAGCAACCACCTGGTTTCGAATCACGAGAGCATCYTGATCATGTGTGCAAGCTGAAAAAGGCCTTGTACGGATT
AAAACAAGCTCCACGAGCTTGGTATGGAAAGGTTGCTCARTTCCTTCAATTTTGCGGCTATGAGGCATCAAATTCTGACCCG
AGTCTATTCTTCAAGAAGAAAGGAGGAGTYCACGTAGTAGTTCTTCTTTACGTGGATGATATGATAATTACYGGGAATGATG
ATGCAGAAATTGCTCGCTTACAAGAAGAYATGTCGATAAGGTTCGAGATGAAGAAGTTGGGTGAGCTGAATAACTTTCTTGG
CCTAGAGGTTGAAAGAGKAAAKGATGGAATATTCGTTGGTCAACAAGGTTATGCAAGAAGGATTGTTGAGAAGTTCGGGGT
GCACGAAGGAAAGAYGCGCACTACTCCGATGGATGTGAACATCAAGCTCAAACGTGATGAAGGTTCATTGTTACCTGATCC
TCGACCTTATCGYGCTCTTGTGGGAAGTCTTCTATACTTAACCATTACAAGACCTGATATTGCCTTCGCRGTAGGTCTKGTA
AGTCGGTTTATGCAAGCACCAAGGAAACCACACTTAGAAGCGGCAAAGAAGATCTTGAAGTATGTCAAGACAACTCTTGAC
ATGGGCTTGGTGTACAAGTACAATGCAAAGATTTCTCTTCATGGTTTTACAGATGCTGACTTTGGTGGAGATCTAGATGACC
GAAAATCAACTTCGGGCTATGTGTTCTTATGTGGAGACACAAGCATTTCGTGGTGTAGCAAGAAACAACCAACGGTATCGCT
ATCTACCACTGAAGCGGAGTATAAAGCTTCCACACTCGCTGCTCAAGAGTGTATATGGCTTCGAAGACTCTTTGAAGATTTG
TTCGAGCCCATAAATAAACCGGTGGCTATCTACGGAGATAATCAAAGTGCTATCAAGTTAGCTAACAATCCGGTGTTTCATG
CAAGGACAAARCATATTGAGTTAGAACATCACTTCATAAGGGAGAAGGTTCTTCTTGATGGAACAATTGAAGCCTTGGAAGT
TCGAAGTGAGGACAACGTTGCAGACATCTTCACCAAGTCATTACCAAAAGGCCAATTCGAGTTACTTCGCTYGAAGCTCGG
GATGGTTGACAAAATCAAGTTTAAGGGGGAGTATTGAAGTTAAACTTGATTTTGGTTTAAGCTTAATTATGGGTTTATAATTC
AATATTCTATATTGGATAAAACCCAAAGTTAAGCCTAAGTTCTAGATTTCTCTAGAGCATCATAGAAAAATCATCACCTCCTTA
AATGTTCTAGAGAATTYTATTAGATAGATATTTAGAWAATTATGTAGAATAATCTAGAAGTTTGTAAGATAAAATCTAGATATT
TTAGTAGAGTTATAGAGATGATGGTAGAATAATCTAGAGACTTTGTAAGCTAACCTTGGAGGCTATAAATACCTCCTCACCC
CCMTCATTTGTAATTWTGTGAGGAAGTTGAACAAGTGAATAAAAAGAAAGTTCAAATAAAAGAACACTCTTTAAAAGTTCTCT
AAGTCTCTCCAAAAGTCTTTCAAGTAGTCTCTAATTGTTCTTCCAAATTTTCTATCATACTTAGAGGRTTTCWTATCTTGATTA
TATCGAGTTTCTCGGGTATTACGGTCTTGGGCTAGTGCTAAGCACTATCGADYTCGGDTTCTTTAACCGAATCA

**Table S3.** List of *Arabidopsis thaliana* accessions negatively tested for presence of *TERESTRA* elements. *Arabidopsis lyrata* MN47 genomic DNA was used as positive control.

| Line | Arabidopsis Biological Resource Centre ID | Line | Arabidopsis Biological Resource Centre ID |
|---|---|---|---|
| Bay-0 | CS76094 | Se-0 | CS76226 |
| Bor-4 | CS76100 | Shahdara | CS76227 |
| Bur-1 | CS76105 | Sq-8 | CS76230 |
| C24 | CS76106 | Uod-7 | CS76296 |
| Ct-1 | CS76114 | Van-0 | CS76297 |
| Oy-0 | CS76203 | Wei-0 | CS76301 |
| Kin-0 | CS76153 | Ws-0 | CS76303 |
| Kno-18 | CS76154 | Yo-0 | CS76305 |
| Ga-0 | CS76133 | Zdr-6 | CS76306 |
| Got7 | CS76136 | Hod | CS76141 |
| Gy-0 | CS76139 | Kbs-Mac-8 | CS76151 |
| Hr-5 | CS76144 | Köln | CS76155 |
| LL-0 | CS76172 | LAC-3 | CS76157 |
| Lp2-2 | CS76176 | LDV-58 | CS76163 |
| Lp2-6 | CS76177 | Liarum | CS76166 |
| Lz-0 | CS76179 | Krot-2 | CS28420 |
| Mr-0 | CS76190 | Li-7 | CS28461 |
| Mt-0 | CS76192 | Mc-0 | CS28490 |
| Nd-1 | CS76197 | Mh-0 | CS28492 |
| Pna-17 | CS76213 | Nc-1 | CS28527 |
| Pro-0 | CS76214 | No-0 | CS28564 |
| Pu2-23 | CS76215 | Udul | CS76269 |
| Ra-0 | CS76216 | UKID101 | CS76270 |
| Ren-1 | CS76218 | UKNW06-436 | CS76278 |
| Rsch-4 | CS76222 | Ull2-3 | CS76293 |

**Figure S8.** The fragment of *TERESTRA* from *A. cebennensis* clone 44. This sequence was identified by BLASTs in NCBI sequence database using *A. lyrata TERESTRA* consensus sequence. Domains were indicated in different colors according to Figure 4A: 5’ LTR (grey), PBS (underlined) and GAG polyprotein (green).

TGAATAATACTGAACGGGTAAACAGAATTATGGTTAAGCTTAATTATGGGTTTATAATTCAATATCCTATATTGA
ACAAAACCCAAAGTTAAGCCAAAGTTCTAGATTTCTCTAGAGCATCATAGAAAAATCATCACCTCCTTAAGTGT
TCTAGAAAATTTTATTAAATAGATATTTAGATAATTATGTAGAATAATCTAGAAGTTTGTAAGATAAAATCTAGAT
ATTTTAGTAGAGTTATAGAGATGATTTTAGAATAATCTAGAGACTTTGTAAGCTAACTTTGGAGGCTATAAATA
CCTCCTCACCCCCTCATTTGTAATCAAGTTGAACAAGTGAATAAAAAGAAAGTTCAAATAAAAGAACCCTCTAT
ATAAATTCTCTAAGTCTCTCTAAAAGTCTTTCAAGTAATCTCTAGTTGTTCTTCCAAATTTTCTATCATACTTAGA
GGATTTCTTATCTTGGTTATATCGAGTTTCTCGGGTATTACGGTCTTGGGCTAGTGCTAAGCACTATCGAGTC
GGTTTCTTTACATGATATCAGAGCCATTCTAATCTTGTGCTCATCAAGATCAAGGTTATAGAGAACTCGGGTTT
ACTCTAAGTATGGAATCTATGGGTCGTGTTGTTGGATTGGGAATGGAGATCTTAAACCAATCTAACTACCGGT
TATGGAAGTCATGTATGGAGTCATACTTGGTTAGTGAGGACTTATGGGATGTCGTCGGCGGAAGTAGTACTT
CACCACCTACCGGAGATGCTGCTACTGCGGAAGCGACAAAGGAGTGGACACGTAAAAATGCCAAGGCGGAG
TTTGCTTTAAAGAGGTCAATATCCTCGGGAGTATTTGAGCATGTCTCAAGGTGTACATCCGCTAGTTCAATTT
GGCAAGCTTTAGATCTACTGTTCAACAAGAAGAACGAAGCTCGTCTATAATTGTTAGAAAACGAGTTGGCGAA
TGCGAAGCTGGGGGAGTCTTCAATCTCGGAGTTTTTCATCAAAGTCAAGAACCTTTGCTCTGAGATCAATAGT
CTTAATCCGGAGGAATCAATCTCGGATGCTCGGTTGAAGCGGTTTATTATTCGAGGTCTTAGACCCGAGTATA
CACCGTTTGTGACTTCGGTTCAAGGATGGGCTACACAACCTTCCTTGGAAGAGTTTGAAAATTTGCTTGCTTC
GCAAGAATCACTAGCGGTACAAATGGCGGGAGTCAAAATTCATGATGACTCGGGGAGCGCTTTTGTAGCTAG
GAGGCAACACAACTTCAAAGCCAAATCCAATGATGGTGGTCCAAGAAAAAATGATGGACATGAAGGATCTTC
TAAGGGAGATAAGAAAAAATTCAAGTGCTATCGGTGTGGAAAACTTGGGCATTTTAAAAAAGATTGCCGAGTC
AAGCTGAAGGAAACAAAGATGGCAAAATCAAAAGGTCATAATGAAGACGAAGAATGGGGAAAATGTTTTATG
GTGGAGGCTGCATCTTCAAGTACATCTACACCTAAGAAGGTGGAAAATAATTGGATCGTAGATTCTGGTTGTA
GCCATCATATCACCGGAGACGAGGAATTATTTTCTAGTCTTCAACGCCATGAAGGAAAAGAAGCAATCATTAC
CGCGAATAATTCAATCCATCACGTTGAGAAGGAAAGAACGGTGGTCATCAAAGGAGATGATGGAAGCCCAAT
CACTCTCGAGAACGTTTACCATGTTCCTGGAGTAAAAAAGAATCTTCTCTCCGTGGTAAATGCGGTGGAGTCA
GGTAACTATGTTTTATTTGGCCCAAGAGATGTTAAATTCTTAAAGAATATTCAAGAGTTAAAGGCGGACATGGT
TCACACTGGAGCACAGGTTAAAGATCTGTACGTTTTATCGGCGTTAAACTCTTATGTAGAGAAGAT

**Figure S9.** *A. halleri TERESTRA* reconstructed based on NCBI BLASTs using *A. lyrata TERESTRA* consensus sequence. Domains were indicated in different colors according to Figure 4A: 5’ LTR (grey), PBS (underlined), GAG polyprotein (green), integrase (violet), reverse transcriptase (yellow), RNAse H1 (blue), PPT (red), 3’ LTR (grey). Note that LTRs could not be reconstructed to a full length.

GGGTTTCTTATCTTGGTTATATCGAGTTTCTCGGATATTACGGTCTTGGGCTAGTGCTAAGCACTATCGAGTC
GGTTTCTTTACATGGTATCAGAGCCATTCTAATCTTGTGCTCTTCAAGATCGGGGTTATAGAGAACTCGGGTT
TACTCTAAGTATGGAATCTATGGGTCGTGTTGTTGGATTGGGAATGGAGATCTTAAACCAATCTAACTACCGG
TTATGAAAGTCATGTATAGAGTCATACTTGGTTAGTGAGGACTTATGGGATGTCGTCGGCGGAAGTAATACTT
CACCACCTACCGGAGATGCTGCAACCGCGGAAGCGACAAAGGAGTGGACACGTAAAAATGCCAAGGCGGA
GTTTGCTTTAAAGAGGTCAATATCTTCGGGAGTATTTGAGCATGTCTCAAGGTGCACATCCGCTAGTTCTATT
TGGCAAGCGTTAGATCGACTGTTCAACAAGAAGAACGAAGCTCGTCTACAATTGTTAGAAAACGAGTTGGCG
AATGCGAAGCAGGGGGAGTCTTCAATCTCAGAGTTTTTCATCAAGGTAAAAAACCTTTGCTCTGAGATCAATA
GTCTTAATCCGGAGGAATCAATCTCGGATGCTCGGTTGAAGCAGTTTATTATTCGAGGTCTACGACCCGAGT
ATACACCGTTTGTGACTTCGGTTCAAGGATGGGCTACACAACCTTCCTTGGAGGAGTTTGAAAATTTGCTTGC
TTCGCAAGAATCACTAGCGGTACAAATGGCGGGAGTCAAAATTCATGATGACTCGGGGAGCGCTTTTGTAGC
TAGGAGGCAACACAACTTCAAAGCCAAATCTAATGATGGTGGTCCAAGAAATAATGATGGACATGAAGGATCT
TCTAAGGGAGATAAGAAAAAATTCAAGTGTTATCGGTGTGGAAAACTTGGGCATTTTAAAAAGGATTGCCGAG
TCAAGCTGAAGGAAACAAACATGGCGGAATCAAAAGGTCATAATGAAGACGAAGAATGAGGAAAATGTTTTA
CGGTGGAGGCTGCATCTTCAAGTACATCTACACCTAAGAAAGTGGAAAATAATTGGATCGTAGATTCTGGTTG
TAGCCATCATATCACTGGAGACGAGAAATTATTTTCTAGTCTTCAACGCCATGAAGGAAAAGAAGCAATCATT
ACCGCAGATAATTCAATCCATCACATTGAGAAGGAAGGAACGGTGGTCATCAAAGGAGATGATGGAAGCCCA
ATCACTCTCGAAAACGTTTACCATGTTCCTGGAGTAAAAAAGAATCTTCTCTCCGTGGTGAATGTGGTGGACT
CAGGTAACTATGTTTTATTTGGCCCAAGGGATGTTAAATTCTTAAAGAATATCCAAGAGTTAAAGGCGGACGC
GCTTCACACTGGAGCACGGGTTAAAGATTTATACGTTTTATCGGCGTCAAACTCTTATGTGGAAAAGATGAGT
ACCAACGATAATGTCTTTATATGGCATGCTAGGCTTAGTCACATAAATAAGACTAAACTCAAGGTTATGGTGAA
TAAAGATTTGGTGAACGGGCTCCCAAAGTTGAAGATCCAAGATGGAGGCACAATTTGTGAAGGATGTCAATA
TGGAAAATCTCATAGGCTTCCATTTGACTATTCCATTTCAAGGTGCAGTTTTCCGTTGGAGAGGGTCCACAGT
GACTTGATGGGTCCGACAAGAACACCCTCATATTCCGGGTATCTCTACATGTTGTTGTTCGTTGACGACTTCT
CTCGGTACACTTGGGTATACTTTGTGAAGGAAAAATCTGAAGTATTCTCCAAGTTTCTAGAATTCAAGGTTACC
GTGGAAGGGGAGTTAGGTCAGAAGATCAAGACTTTAAGGACGGATAATGGAGGGGAGTTCATGTCTAAGGA
GTTTCTCTCTTTTTGTCGAGATAATGGTATCAAGAGAGAATTTACATGTCCGCATACACCTCAACAAAATGGAG
TAGCAGAGAGGAAGATCAGACATCTAAGTGAGACGTGTAGAAGTTGGCTCCATGGAAAAGATTTGCCTAAGG
CGTTATGGGCGGAAGGCATGAGATGTGCAGCTTATGTCATCAACCGGATGCCACTTAGTCCAAACAATATGA
AGTCTCCTTATGAGATGGTTCATGGGAAGAAGCCAACGGTGAAACATCTCAGGATATTTGGATCAGTCTGCTT
CGTCCATGTGTTTGATTCACAAAGAACCAAGTTGGAGGCAAAGGCGAAAAAATGCATATTTGTCGGCTACGAT
GAACAAAGGAAGGGTTGGAGGTGTATGGATTCCGAGACACACAAGTATGTTGTGTCTCGTGATGTTGTCTTT
GATGAAGTTTCTTCATACTACGGGTCACCTCAAGTCTTGGTTGAGAAAGATGGTGCTAATTCCTCTAAGAACG
ATGAATCAACTCTCCAAGTACCATGTGAGAGTGGAAGTCCTGAAAATAAAATTCAAGGTGAAAAGGGGAGCA
CTAACCAAGAAGAAGAAGAGGAACAAGATCATGGTTCGATGGTCAATCAACGGTCGAAAAGAAACATTGTCA
AGCCTGCAAGATATAGAGATGAAGTATTCATCACTACTTATTCATGCTTCTTCACAGCTCCTCTAGATGATGAT
GAGCCATCTTCTTATGATGAAGCTAAAGGAGTTCAAGAATGGGAAGTTGCAATGAAGGAGGAGATGAGTGCT
CTCAAGAAAAATGAAACTTGGCATCTGGTTCCAAAACCCAAGGATGTTGAGCCCGTTTCTTGCAAATGGGTGT
ATCGAATTAAGCAAAAAGCGGATGAGAGCATTGATAGGTTTAAAGCAAGGTTGGTTGCTCGGGGATTCTCTC
AAAAATATGGAGAAGATTATGATGAGACATTTAGTCCTGTGGCGAAGATGACAACTTGATGTGAAGAATGCTT
TTCTATATGGTGAGCTTGATAAAAGTATCTTCATGGAGCAACCACCTGGTTTCGAATCGCGAGAGCATCCTGA
TCATGTGTGCAAGCTGAAAAAGGCCTTGTACGGATTAAAACAAGCTCCACAAGCTTGGTATGGAAAGGTTGC
TCAATTCCTTCAATTTTGTGGCTATGAGGCATCAAATTCTGACCCGAGTCTATTCTTCAAGAAGAAAGGAGGA
GTCCACGTAGTAGTTCTTCTTTACGTGGATGATATGATAATTACCGGGAATGATGATGCAGAAATTGCTCGCT
TACAAGAAGACATGTCGATAAGGTTCGAGATGAAGAAGTTGGGTGAGCTGAATAACTTTCTTGGCCTAGAGG
TTGAAAGAAGAAAGGATGGAATATTCGTTGGTCAACAAGGTTATGCAAGAAGGATTGTTGAGAAATTCAGGGT
GCACGAAGGAAAGACGCGCACTACTCCGATGGATGTGACCATCAAGCTCAAACGTGATGAAGGTTCATTGTT
ACCTGATCCTCGACCTTATCGCGCTCTTGTGGGAAGTCTTCTATACTTAACCATTACAAGACCTGATATTGCC
TTCGCAGTAGGTCTGGTAAGTCGGTTTATGCAAGCACCAAGGAAACCACACTTAGAAGCGGCAAAGAAGATC
TTGAAGTATGTCAAGACAACTCTTGACATGGGCTTGGTGTACAAGTACAATGCAAAGATTTCTCTTATTGGTTT
TACGGATGCTGACTTTGGTGGAGATCTAGATGACCGAAAATCAACTTCGGGCAATGTGTTCTTATGTGGAGA
CACAAGCATTTCGTGGTGTAGCAAGAAACAACCAACGGTATCGCTATCTACCACTGAAGCGGAGTATAAAGC
TTCCACACTCGCTGCTCAAGAGTGTAAATGGCTTCGAAGACTCTTTGAAGATTTGTTCGAGCCTATAAATAAA
CCGGTGGCTATCTACGGAGATAATCAAAGTGCTATCAAGTTAGCTAACAATCCGGTGTTTCATGCAAGGACAA
AGCATATTGAGTTAGAACATCACTTCATAAGGGAGAAGGTTCTTGATGGAACAATTGAAGCCTTAGAAGTTCG
AAGTGAGGACAACGTTGCGGACATCTTCACCAAGTCATTACCAAAAGGCCAATTCGAGTTACTTCGCTCGAA
GCTCGGGATGGTTGACAAAATCAAGTTTAAGGGGGAGTATTGAAGTTAAACTTGATTTTGGTTTAAGCTTAAT
TATGGGTTTATAATTCAATATTCTATATTGGACAAAACCCAAAGTTAAGCCTAAGTTCTAGATTTCTCTAGAGC
ATCATAGAAAAATCATCACCTCCTTAAATGTTGAACAAGTGAATAAAAAGAAAGTTCAAATAAAATTAAAGAAC
ACTCTTTAAAAGTTCTCTAAGTCTCTCCAAAAGTCTTTCAAGTAATCTCTAATTGTTCTTCCAAATTTTCTATCAT
ACTTAGA

**Figure S10.** Heat responsive elements (HREs) found in 5' long terminal repeats of *TERESTRA* elements. All identified *TERESTRA* elements (irrespective of their heat-responsiveness) are shown. HRE types: red – 4P, yellow – gap, green – step.

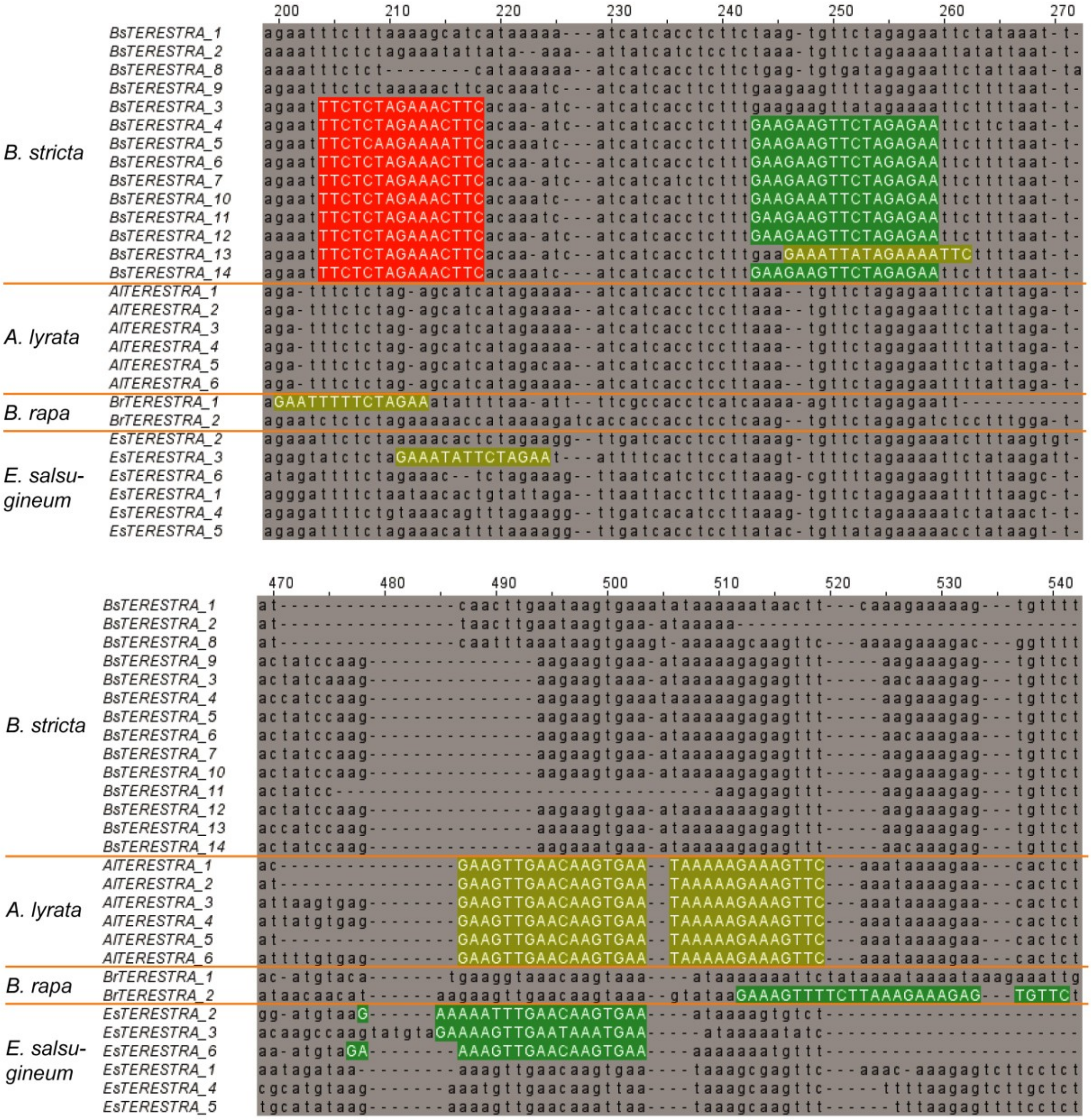

**Figure S11.** Transcriptional response of *ONSEN*, *COPIA37* and *TERESTRA* to DNA methylation inhibitor treatments in *A. lyrata*. Relative transcript accumulation after 48 h control, 10 μM 3-deazaneplanocin A (DZNep) and 40 μM zebularine treatment. Transcript amounts were normalized to *UBC28*. Error bars indicate variation between two biological replicates and * shows statistically significant differences at $\alpha = 0.05$ in Student's T-test.

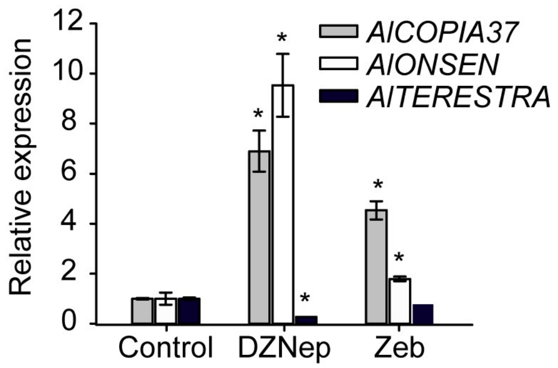

**Figure S12.** Density of RNA-sequencing reads mapping over *APUM9 – ROMANIAT5-2* region.

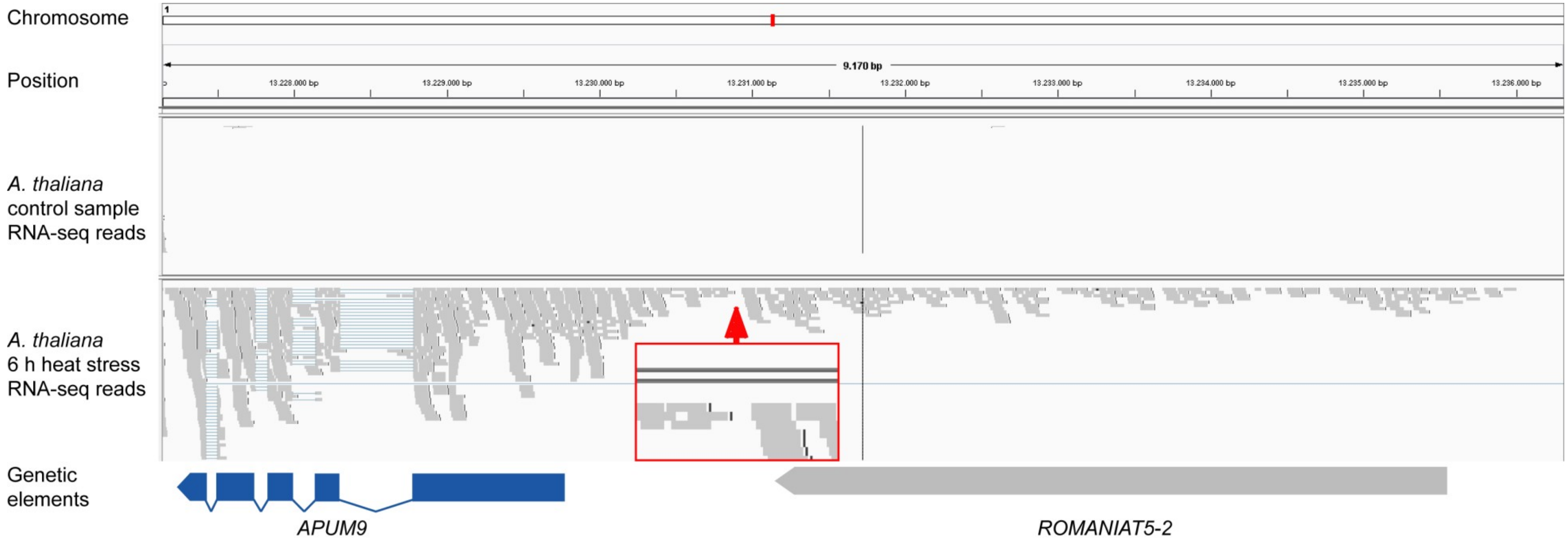

**Figure S13.** Putative heat responsive elements (HREs) in 5'/3' long terminal repeats of *ROMANIAT5* elements. HRE types: orange – 3P, yellow – gap, green – step.

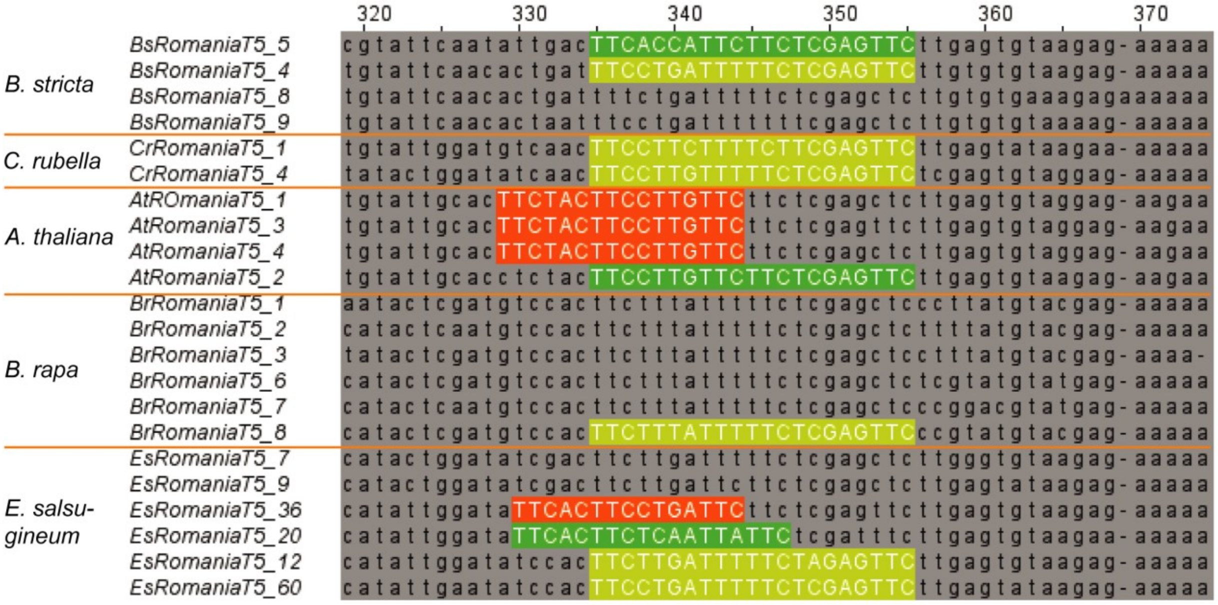

**Table S4.** Primers used in this study.

| | | | | |
|---|---|---|---|---|
| *At - Arabidopsis thaliana* | | | | |
| *Al - Arabidopsis lyrata* | | | | |
| *Ba - Ballantinia antipoda* | | | | |
| *Bs - Boechera stricta* | | | | |
| *Br - Brassica rapa* | | | | |
| *Cr - Capsella rubella* | | | | |
| *Es - Eutrema salsugineum* | | | | |
| | | | | |
| Target | Species | Primer name | Sequence (5' to 3') | Application |
| *COPIA20* | *At, Al* | C20_qF2 | TACATGAAGCCACCACCGGGT | RT-qPCR |
| | *At, Al* | C20_qR3 | TCATCTCCGGGAATGACAAGGTA | RT-qPCR |
| *COPIA37* | *At* | AtC37_F2 | AGCTTAACTACAGAAGGGAAGGA | RT-qPCR |
| | *At* | AtC37_R2 | CTCTCCAATCTCTCATTTTCTCG | RT-qPCR |
| | *Cr* | CrC37_F1 | GGAGGTAGGTGAGACAAGACA | RT-qPCR |
| | *Cr* | C37_LTRR1 | AACCGCTACGTCTCGGGG | RT-qPCR |
| | *Al* | AlyC37qF3 | AACGCAGCCGAAGCTAATC | RT-qPCR |
| | *Al* | AlyC37qR3 | CTCCATCACCGGCTAACAAC | RT-qPCR |
| | *Br* | EsC37-u_F1 | ACAGGTGGGyCTTTAATGGGC | RT-qPCR |
| | *Br, Es* | C37_u_LTRR1 | AACCGSTACGTCTCGGGG | RT-qPCR |
| | *Es* | EsC37_F1 | ACAGGTGGGTCTTTAATGGGC | RT-qPCR |
| *COPIA78* | *At, Bs* | AtCOPIA78qF2 | CGGTGCTCACAAAGAGCAACTATG | RT-qPCR |
| | *At, Bs* | AtCOPIA78qR3 | ATCCTTGATAGATTAGACAGAGAGCT | RT-qPCR |
| | *Al* | AlCOPIA78qF3 | ACAATGCTCACAAAGAGCAACTATG | RT-qPCR |
| | *Al* | COPIA78qR3 | ATCCTTGATAGATTAGACAGAGAGCT | RT-qPCR |
| | *Ba* | Ba_ONS_F1 | ACATGTACCGGATGAGAAGCG | RT-qPCR |
| | *Ba* | Ba_ONS_R1 | TGTGTCCGGGTTGTAGAGCT | RT-qPCR |
| | *Br* | Br_ONS_F1 | TTGCGTGCATGATGTCAGGT | RT-qPCR |
| | *Br* | Br_ONS_R1 | ACGCCAATGGAATGTCGAGT | RT-qPCR |
| | *Es* | EsONS_F5 | GTCTTGGCTTTGGCATCTTC | RT-qPCR |
| | *Es* | EsONS_R5 | GCGCTCTTGTTTCTGACTCC | RT-qPCR |
| *GAPC-2* | *At, Al, Ba, Bs, Br, Cr, Es, Aa* | GAPC-2_F | ATCGGTCGTTTGGTTGCTAGAGT | RT-qPCR |
| | *At, Al, Ba, Bs, Br, Cr, Es, Aa* | GAPC-2_R | ACAAAGTCAGCTCCAGCCTCA | RT-qPCR |
| *GFP-4* | *At* | GFP4-F | GGCACGACTTCTTCAAGAGC | RT-qPCR |
| | *At* | GFP4-R | AAAGGGCAGATTGTGTGGAC | RT-qPCR |
| *TERESTRA* | *Bs, Br, Es* | TERESTRA_F1 | TCATGTATGGAGTCATACTTGGT | RT-qPCR |
| | *Bs, Br, Es* | TERESTRA_u_R1 | CACCTTGAGACATGCTCAAATA | RT-qPCR |
| | *At, Al* | TERESTRAF2 | GTATTACGGTCTTGGGCTAGTG | RT-qPCR |
| | *At, Al* | TERESTRA_R2 | ACCAAGTATGACTCCATACATGAC | RT-qPCR |
| *HSP101* | *At* | HSP101qF | TGAGCTAGCTGTGAATGCAGGACATGCTC | RT-qPCR |

| | | | | |
|---|---|---|---|---|
| | *At* | HSP101qR | ATCACTCTTTCAGCAGATTGAGCTGCGTT | RT-qPCR |
| *ROMANIAT5* | *At, Al, Br, Bs, Cr, Es* | RT5_gener_F2 | CGTGGAGGGATATGCTTCGTT | RT-qPCR |
| | *At, Al, Br, Bs, Cr, Es* | RT5_gener_R2 | TGCAATCTCGTGGCGTTCT | RT-qPCR |
| | *At* | ROMANIAT5-2qF | TTGTCACTTGAACGAATGTATTGCACC | RT-qPCR |
| | *At* | ROMANIAT5-2qR | GCCAACAAGAATATATGAAGATAATGC | RT-qPCR |
| | *At* | ROM_sense | TTCCAATGCTTTGAACATGA | cDNA synthesis |
| | *At* | ROM_antiS | TGAATACATCACGAGAAAAGA | cDNA synthesis |
| *PUM9* | *At* | PUM9qF | CATGGTTCCCATTCAGATCTTTGACA | RT-qPCR |
| | *At* | PUM9qR | TTTAGTCAACAAGTCCACGAGCTCA | RT-qPCR |
| | *At* | PUM9_sense | TCACGTGGCTACTAAACTTCT | cDNA synthesis |
| | *At* | PUM9_antiS | AGCTTAACTTCCGAGGAG | cDNA synthesis |
| *UBC28* | *At,Al, Ba, Bs, Br, Cr, Es, Aa* | UBC28qF | TCCAGAAGGATCCTCCAACTTCCTGCAGT | RT-qPCR |
| | *At, Al, Ba, Bs, Br, Cr, Es, Aa* | UBC28qR | ATGGTTACGAGAAAGACACCGCCTGAATA | RT-qPCR |
| *18S* | *At* | 18S-1062-F | TTATAGGACTCCGCTGGCAC | RT-qPCR |
| | *At* | 18S-1345-R | CAGGCTGAGGTCTCGTTCAT | RT-qPCR |
